# High-Sensitivity and High-Precision Near-Infrared Luminescence Thermometry Using Mn^5+^-Activated Sr_5_(VO_4_)_3_Cl and Ba_5_(VO_4_)_3_Cl-Based Phosphors

**DOI:** 10.1021/acs.chemmater.6c00822

**Published:** 2026-07-08

**Authors:** Abbi L. Mullins, Artemijs Krimovs, Zoran Ristić, Vesna Djordjević, J. A. Gareth Williams, Miroslav D. Dramićanin, Ivana Radosavljević Evans

**Affiliations:** † Department of Chemistry, 3057 Durham University, Lower Mountjoy, Durham DH1 3LE, U.K.; ‡ Centre of Excellence for Photoconversion, Vinča Institute of Nuclear Sciences National Institute of the Republic of Serbia, University of Belgrade, P.O. Box 522, Belgrade 11001, Serbia

## Abstract

A series of near-infrared-emitting materials of composition A_5_(V_1–*x*_O_4_)_3_Cl:Mn_*x*_ (A = Ca^2+^, Sr^2+^, Ba^2+^; *x* = 0.00, 0.01, 0.03, 0.05) were prepared and characterized by X-ray diffraction, solid-state NMR, and room-temperature luminescence measurements. Two candidates (Sr_5_(V_0.97_O_4_)_3_Cl:Mn_0.03_ and Ba_5_(V_0.97_O_4_)_3_Cl:Mn_0.03_) were selected for detailed evaluation as luminescence thermometers. Three single-parameter luminescence thermometry methods (based on relative intensities, band positions, and band widths) were explored. Through multiparametric thermometry, we show that Sr_5_(V_0.97_O_4_)_3_Cl:Mn_0.03_ has remarkable performance parameters at physiological temperature (308 K): temperature relative sensitivity *S*_r_ = 3.14% K^–1^ and temperature resolution of 0.042 K. The multiparametric approach gives even better accuracy than the best individual readout (that based on luminescence intensity ratio, LIR), while retaining the exceptionally good temperature determination precision of the best single-parametric method (that based on bandwidth). Three different parameter-free full-spectrum luminescence thermometry methods were also applied, giving temperature readout accuracy values ranging from 0.03 to 0.08 K, while maintaining average resolution values of 0.07–0.08 and 0.07 K across the temperature range studied. These performance parameters – coupled with the remarkable brightness in the second biological window (photoluminescence quantum yield of 47%) and the thermal stability at both long-range and local-structure length-scales demonstrated by variable-temperature X-ray diffraction and solid-state NMR, respectively—make Sr_5_(V_0.97_O_4_)_3_Cl:Mn_0.03_ a very promising candidate for luminescence thermometry at physiological temperatures.

## Graphical abstract

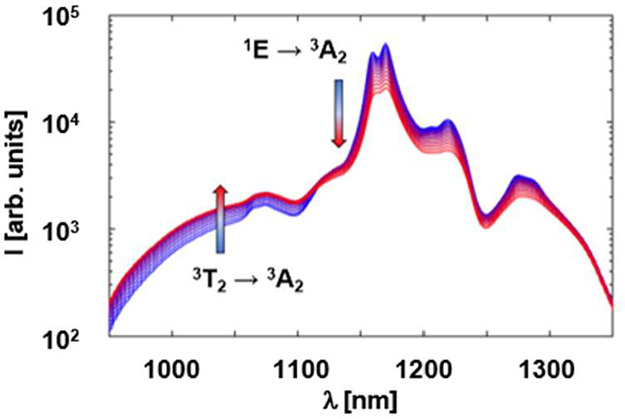

## 1 Introduction

Luminescence thermometry is an important area of fundamental and applied research. The method is enabled by changes in the optical properties of luminescent materials with temperature.^1^ It is especially advantageous in hard-to-access environments since, although the emitter itself must make thermal contact with the sample investigated, the readout may be undertaken remotely.^2–5^ This allows for temperature measurements in, for example, microfluidic or nanoscale environments, including biological samples^6–8^ and in vivo applications. The spectral region from 650 to 1800 nm is of interest since absorption, autofluorescence, and scattering by tissue are reduced in this region compared to shorter wavelengths.^9^ The 1000–1350 nm range (the so-called “second biological window” or BW-2) is of particular importance as it allows the deepest penetration of skin and tissue.^10^

Luminescence thermometry can make use of various changes in the emission parameters with temperature, including the absolute intensity at a single wavelength, the relative intensity at two or more wavelengths, the emission band position (λ_max_ value), the width of the emission band (usually quantified in terms of the full-width at half-maximum or fwhm), and the rate of decay of the light (normally quantified in terms of the lifetime τ, requiring either a pulsed or intensity-modulated excitation source to measure it).^11^ The luminescence intensity ratio (LIR) method, which makes use of changes in the relative intensity at two or more wavelengths, is particularly attractive, as it is a self-referencing method that is not compromised by fluctuations in the efficiency of light delivery or collection.^12^ It requires either a single luminescent center with thermally coupled excited states that are in a Boltzmann-type equilibrium on a time scale comparable to the rate of radiative decay (*k*_r_) of the lower state, or multiple luminescent centers with spectrally well-separated emission bands.^13,14^ Lanthanide ions (Ln^3+^) are well-established as luminescent centers for thermometry applications owing to their numerous emissive and inherently narrow f–f transitions, many of which fall within at least one of the biological windows.^15–20^ The relative sensitivity of LIR thermometry, *S*_r_ (a measure of the material’s change in luminescence property with temperature) based on a single Ln^3+^ activator is, however, limited to ∼2.8% K^–1^ at 300 K.^21^ Higher sensitivities can be achieved by introducing a second lanthanide or transition metal (TM) ion.^22^ Alternatively, two or more TM ions can be used. In contrast to the contracted 4f orbitals, which render Ln^3+^ emission rather independent of local environment, the more diffuse d-orbitals in TMs are highly sensitive to the crystal field. Excitation and emission from luminescent TM centers can therefore be tuned through careful selection of the host material.^23,24^ In the appropriate environment, Ni^2+^, Cr^4+^, and Mn^5+^ ions can emit in the desired biological window.^25–28^

While *S*_r_ is generally seen as the most important figure of merit for luminescence thermometry, the uncertainty, σ_r_, in the measurement of the emission intensity is of paramount importance for practical performance. Together, these two parameters determine the temperature resolution of the luminescence thermometer, δ*T*. The ideal luminescence thermometry phosphor should have a high quantum yield and a large molar extinction coefficient at the excitation wavelength λ_ex_ to ensure high brightness. NIR phosphors tend to suffer from low quantum yields due to the small energy gap between the excited state and the ground state, resulting in significant nonradiative relaxation through vibrational pathways. Furthermore, Ln^3+^ ions have the inherent disadvantage of low molar extinction coefficients, making TM ions—especially when tetrahedrally coordinated, with their associated larger molar extinction coefficients—particularly attractive. Mixed metal oxides containing tetrahedral Mn^5+^ have been the subject of research interest for NIR laser applications based on the narrow 1E → 3A_2_ emission of the d^2^ ion.^29–31^ In the pursuit of new blue and blue-green pigments, MnO_4_^3–^ tetrahedra have been identified as potential chromophores, owing to their absorption in the red-orange regions of the electromagnetic spectrum and the increase in absorption at 450 nm as Mn^5+^ content increases, causing a shift to greener hues.^32,33^ Efforts have recently been made to exploit this ion for luminescence thermometry via LIR, lifetime, and band-shift readout methods.^28,34–38^

The host material for the design and development of Mn^5+^-based luminescence thermometers must meet several requirements: (1) it must be chemically and crystallographically suitable to stabilize Mn^5+^ ions in tetrahedral or pseudotetrahedral environments; (2) its bandgap must be sufficiently large to accommodate the Mn^5+^ energy levels associated with the key transitions; and (3) it must be chemically and thermally stable under the conditions relevant to particular applications. Apatite-related materials are known both as natural biomaterials (e.g., in bone and teeth) and as synthetic functional materials with a range of technological applications.^39–46^ Owing to the compositional flexibility captured by the general formula A_10_(TO_4_)_6_X_2±*x*_ (where A = alkaline earth or rare earth metal; T = tetrahedrally coordinated cation (e.g., P^5+^, V^5+^, Si^4+^, Ge^4+^); X = halide, O^2–^ or OH^–^), and the corresponding crystallographic flexibility, the apatite structure is well-suited for the development of phosphors. The ideal apatite can be described as a framework in which columns of face-sharing AO_6_ trigonal prisms share corners with TO_4_ tetrahedra (dark blue and orange polyhedra, respectively, in Figure 1a), forming channels parallel to the *c*-axis, in which additional A cations and the X anions are located (light blue and green/gray spheres in Figure 1). The space group adopted is usually hexagonal *P*6_3_/*m*, with two unique A sites and one unique T site. Low-symmetry apatites are less common, and typically crystallize in space group *P*-1 with five unique A sites and two independent T sites, providing even more possibilities to fine-tune the phosphor properties.^47,48^ An additional advantage of the apatite structure is that it readily accommodates mixed anions. While oxides are the most widely researched phosphor hosts historically, mixed anion oxyhalides are very attractive hosts that have been explored for lighting phosphors, laser materials, white light-emitting diodes (LEDs), and luminescence thermometry, with performance improvements over purely oxide-based or purely halide-based hosts.^49–58^

**Figure 1:**
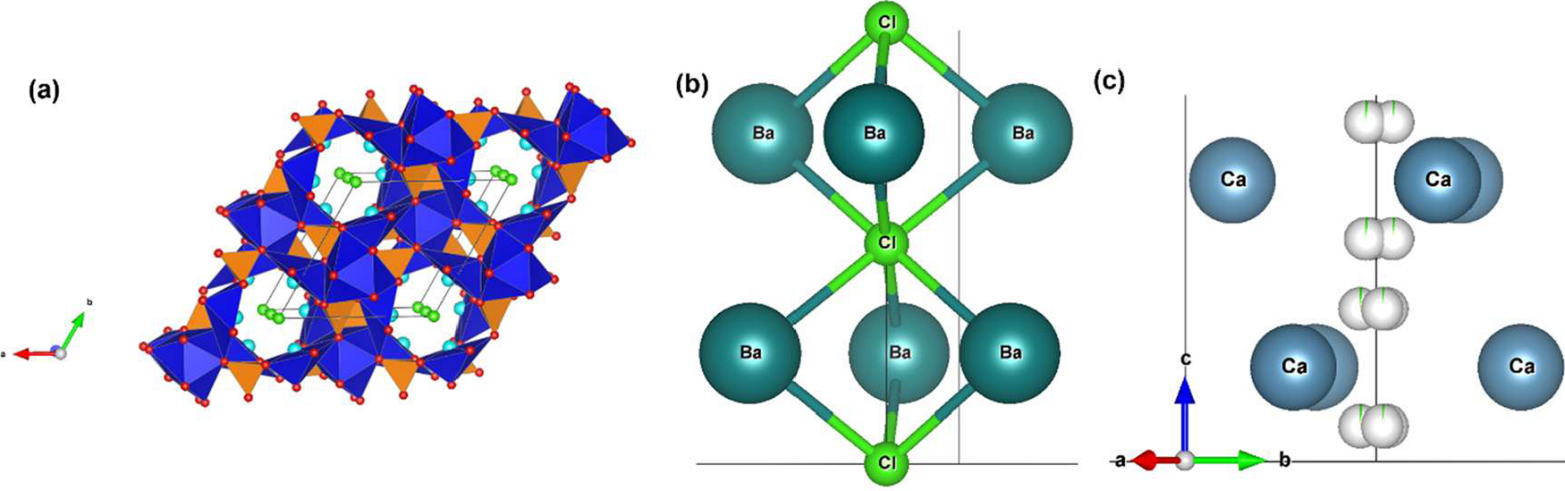
(a) View of the A_10_(TO_4_)_6_X_2_ apatite structure in (approximately) the (*ab*) plane. Dark blue polyhedra and light blue spheres represent two crystallographically unique A atoms; orange tetrahedra represent TO_4_ groups; green spheres represent X anions. (b) Crystallographically ordered distribution of Cl^–^ ions (light green spheres) in Ba^2^^+^ and Sr^2+^-containing materials. (c) Disordered distribution of Cl^–^ ions (gray spheres with some light green, indicating partial occupancy) in Ca^2+^-containing materials.^65^

In this work, we investigated a series of apatite-type Mn^5+^-doped A_5_(VO_4_)_3_Cl oxychlorides (A = Ca^2+^, Sr^2+^, Ba^2+^). Following the initial conventional solid-state syntheses, which produced samples containing small amounts (1–4 wt %) of impurities and screening of room-temperature luminescence, two candidates, Sr_5_(V_0.97_O_4_)_3_Cl:Mn_0.03_ and Ba_5_(V_0.97_O_4_)_3_Cl:Mn_0.03_, were selected for further work: sol–gel syntheses to produce pure samples and extensive characterization for NIR luminescence thermometry application. Vanadate hosts were chosen owing to the match between the ionic radii of V^5+^ and Mn^5+^ (0.355 and 0.33 Å, respectively^59^). The thermal stability at average and local structural length-scales was demonstrated using variable-temperature powder X-ray diffraction and solid-state nuclear magnetic resonance spectroscopy, respectively. Variable-temperature luminescence emission data for the ^3^T_2_ and 1E emissions in the temperature range 308–373 K were analyzed for LIR performance, as well as band-shift and bandwidth-based thermometry possibilities. Furthermore, multilinear parametric regression (MLR) was performed for Sr_5_(V_0.97_O_4_)_3_Cl:Mn_0.03_, leading to a higher *S*_r_ than that possible by any single readout method alone.

## 2 Results and Discussion

### 2.1 Structural Characterization

The samples prepared initially by the conventional solid-state method and subsequently by the sol–gel route were characterized by PXRD to establish the phase identity and purity. The PXRD patterns of the host materials and phosphors of general formula A_5_(V_1–*x*_O_4_)_3_Cl:Mn_*x*_ (A = Ca^2+^, Sr^2+^, Ba^2+^; *x* = 0.00, 0.01, 0.03, 0.05) prepared by the solid-state route were analyzed by the Rietveld method using structural models from the literature (fits shown in Figures S1–S3).^60–62^ These samples contained 1 to 4 wt % impurities, identified as Sr_3_(VO_4_)_2_, Ba_3_(VO_4_)_2_, Ca_2_MnO_4_, and CaMnO_3_, which were included as additional phases in the Rietveld fitting.^63,64^ These samples were used only for screening purposes (room temperature luminescence measurements), upon which two materials, Sr_5_(V_0.97_O_4_)_3_Cl:Mn_0.03_ and Ba_5_(V_0.97_O_4_)_3_Cl:Mn_0.03_, were selected for further work: preparation of pure samples by sol–gel methods and detailed variable-temperature characterization.

All target materials investigated crystallize in the hexagonal space group *P*6_3_/*m*, with the Mn^5+^ ions replacing V^5+^ on the 6h Wyckoff site with local symmetry *C*_s_ (centers of the orange tetrahedra in Figure 1a). The key structural difference between A = Ca^2+^, Sr^2+^, Ba^2+^ compounds observed by PXRD is that the Sr^2+^ and Ba^2+^ materials are fully ordered crystallographically, while the Ca^2+^ materials contain Cl^–^ ions disordered over partially occupied sites in the channels parallel to the *c*-axis (Figure 1b,c, respectively).

The Cl^–^ anion disorder in the channels, which affects the local environment of the cations occupying tetrahedral sites, was also evidenced by solid-state NMR (Section 2.2). The same models were used for the detailed Rietveld analysis of Sr_5_(V_0.97_O_4_)_3_Cl:Mn_0.03_ and Ba_5_(V_0.97_O_4_)_3_Cl:Mn_0.03_ prepared by the sol–gel method, with excellent agreement obtained for both PXRD patterns, and no peaks unaccounted for by the target phase. These phase-pure samples were used for detailed physical property measurements. Figure S5 shows the homogeneous distribution of Mn^5+^ in Sr_5_(V_0.97_O_4_)_3_Cl:Mn_0.03_ and Ba_5_(V_0.97_O_4_)_3_Cl:Mn_0.03_ by EDX chemical mapping. For Sr_5_(V_0.97_O_4_)_3_Cl:Mn_0.03_, the agreement factor obtained was *R*_wp_ = 3.20% (Rietveld fit shown in Figure 2a).

**Figure 2:**
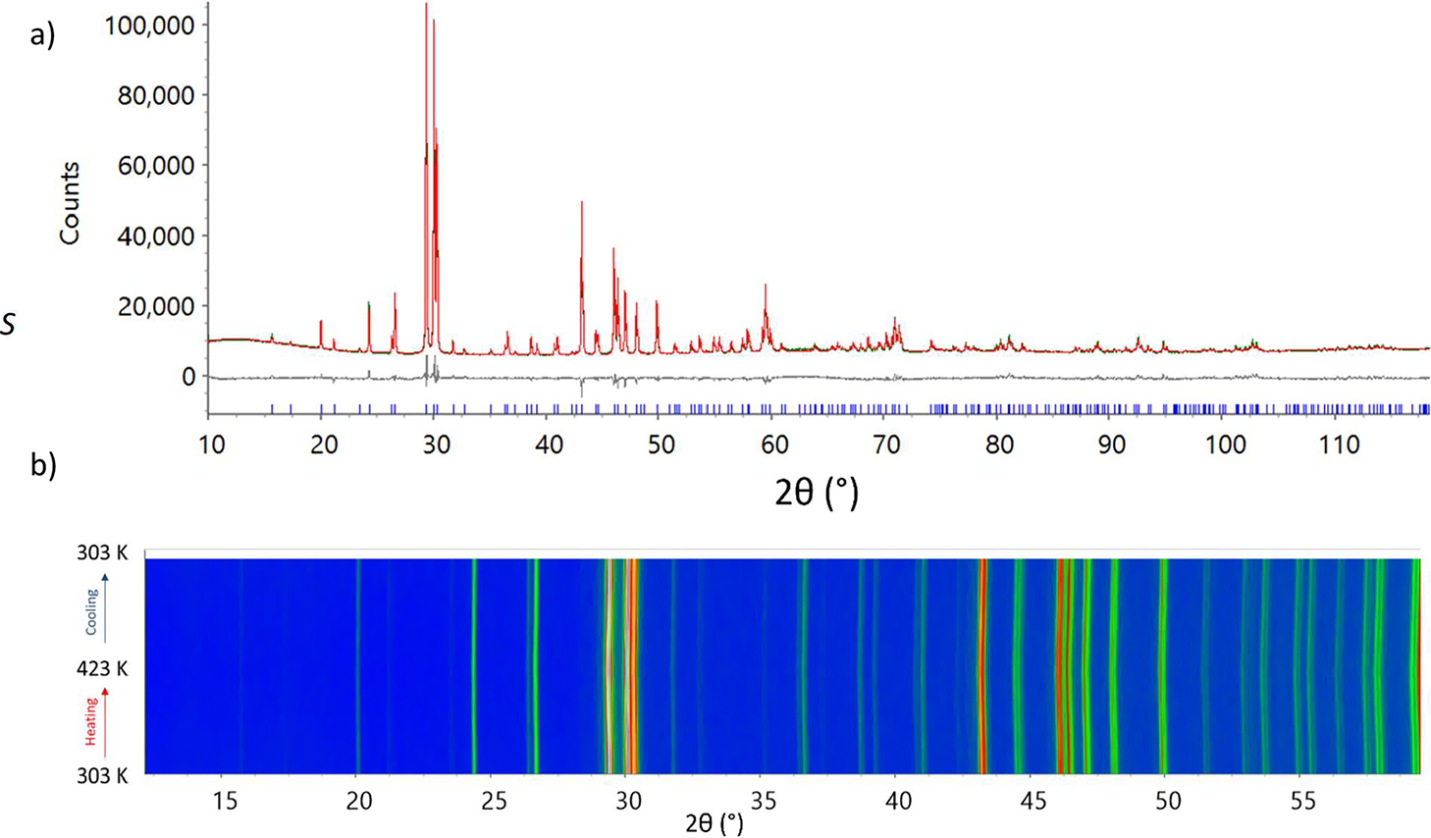
(a) Final Rietveld fit for Sr_5_(V_0.97_O_4_)_3_Cl:Mn_0.03_ (*R*_wp_= 3.20%). The green curve shows the experimental data, the red curve shows the calculated pattern, and the difference curve is depicted in gray. Tick-marks represent the Bragg peak positions. (b) Variable-temperature PXRD data collected on Sr_5_(V_0.97_O_4_)_3_Cl:Mn^5+^_0.03_ on heating and cooling.

The unit cell parameters determined for Sr_5_(V_0.97_O_4_)_3_Cl:Mn_0.03_ are *a* = 10.20911(9) Å, *c* = 7.30625(7) Å, *V* = 659.48(1) Å^3^; the average Sr_5_(V_0.97_O_4_)_3_Cl:Mn_0.03_ V–O bond length is 1.721(5) Å, while the O–V–O bond angles range from 105.4(5)° to 113.0(5)°. The bond lengths give a V^5+^ bond valence sum of 5.02(7). Details for the isostructural Ba_5_(V_0.97_O_4_)_3_Cl:Mn_0.03_ can be found in Figure S4a and Table S2. We note that material with this nominal composition has recently been reported; however, the PXRD pattern shown contains a number of Bragg peaks unaccounted for by the apatite phase, indicating that the sample used for the reported physical property measurements was not pure.^66^

Variable-temperature PXRD data on Sr_5_(V_0.97_O_4_)_3_Cl:Mn_0.03_ show smooth thermal expansion trends, demonstrating thermal stability and absence of structural phase transitions in the temperature range of interest (Figure 2b, with similar data for the Ba-material shown in Figure S4b). Unit cell parameters extracted from Rietveld refinements were used to determine the thermal expansion coefficient:

$$\alpha_{V}=\frac{1}{V}\frac{\partial V}{\partial T}$$ 1

where *V* is the unit cell volume and *T* is temperature. The volume expansion coefficient is ∼4.0 × 10^–5^ K^–1^, comparable to values reported for similar apatite-type materials.^45,62,67^

### 2.2 Investigation of Local Structure by ^51^V Solid-State NMR

To probe the geometry of tetrahedral crystallographic sites in more detail, ^51^V ssNMR experiments were performed using ^51^V as a proxy for the local environments of the Mn^5+^ ions doped onto these crystallographic sites. The spectra obtained for six samples prepared by the solid-state method (three hosts and three phosphors containing 3% Mn^5+^) are shown in Figure S6. The spectra for the Ca-containing pair reflect a broad range of ^51^V environments (Figure S6a,b), presumably associated with local structure distortions arising from the crystallographic disorder in the channels described in Section 2.1 and Figure 1c. The Sr_5_(VO_4_)_3_Cl host shows a single ^51^V resonance at – 609 ppm (Figure S6c), characteristic of pseudotetrahedral ^51^V coordination environments, with a weak satellite to the left of the central transition, consistent with the quadrupolar nature of ^51^V.^68^ In addition to the resonance at −609 ppm, the spectrum of Sr_5_(V_0.97_O_4_)_3_Cl:Mn_0.03_ contains a very low-intensity broad feature at −574 ppm (Figure S6d). To test whether this feature arises from the low-level impurity Sr_3_(VO_4_)_2_ identified by PXRD, ^51^V ssNMR spectra were recorded on pure Sr_3_(VO_4_)_2_, which showed a single resonance at −611 ppm (Figure S7a). Therefore, the weak broad feature at −574 ppm observed in the spectrum of Sr_5_(V_0.97_O_4_)_3_Cl:Mn_0.03_ does not originate from the impurity. Instead, it represents a small fraction of the V^5+^ environments distorted by the proximity of Mn^5+^ dopants. The same feature is observed for Ba_5_(V_0.97_O_4_)_3_Cl:Mn_0.03_ (Figures S6e,f and S7b).

Variable-temperature ^51^V ssNMR spectra show a gradual upfield shift of the main resonance on heating, amounting to a total shift of about 5 ppm over the temperature range observed, consistent with the thermal expansion of the material and the absence of significant or abrupt local structure changes. This demonstrates the stability of the local coordination environments of the Mn^5+^ ions in Sr_5_(V_0.97_O_4_)_3_Cl:Mn_0.03_ and Ba_5_(V_0.97_O_4_)_3_Cl:Mn_0.03_ over the temperature range (Figure 3).

**Figure 3:**
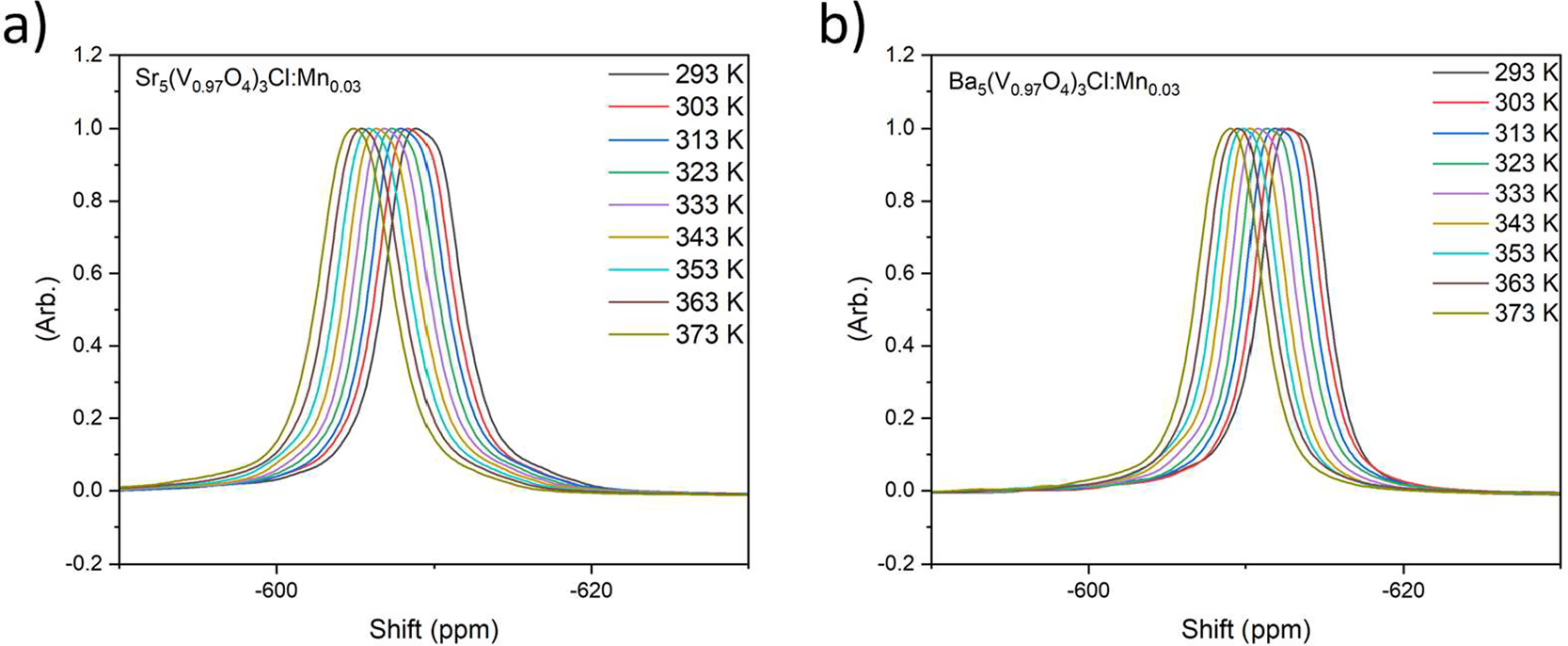
Solid-state ^51^V NMR spectra collected between 20 and 100 °C on (a) Sr_5_(V_0.97_O_4_)_3_Cl:Mn_0.03_ and (b) Ba_5_(V_0.97_O_4_)_3_Cl:Mn_0.03_.

### 2.3 Room-Temperature Photoluminescence

The room-temperature emission spectrum of Sr_5_(V_0.97_O_4_)_3_Cl:Mn_0.03_ obtained on excitation at 700 nm is shown in Figure 4b.

**Figure 4:**
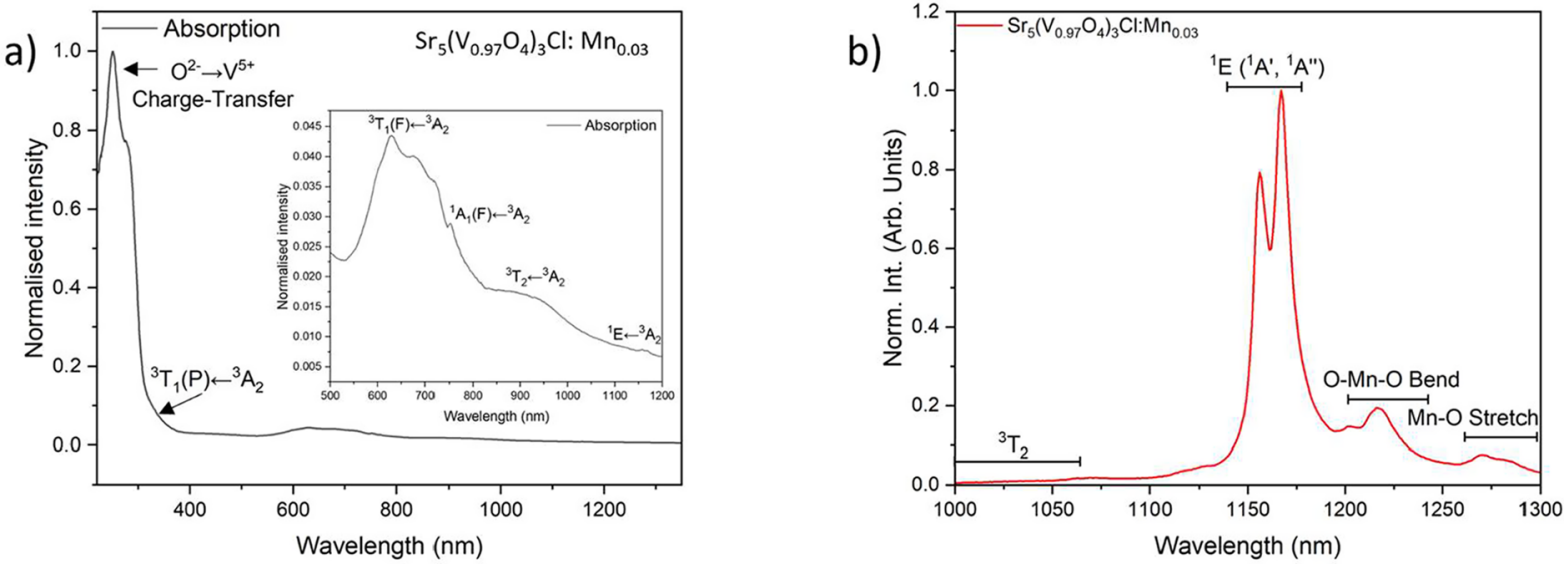
(a) Normalized room-temperature absorption (black curve obtained from diffuse reflectance) and expansion of the 500–1200 nm region of the pseudoabsorption spectrum of Sr_5_(V_0.97_O_4_)_3_Cl:Mn_0.03_ (inset). (b) Emission spectrum of Sr_5_(V_0.97_O_4_)_3_Cl:Mn_0.03_, excited at 700 nm.

The emission spectrum is dominated by the band at 1167 nm, arising from the spin-forbidden transition from the Mn^5+^1E excited state to the 3A_2_ ground state. The band is split into two components (separation of 11 nm or 82 cm^–1^), as the Mn^5+^ ions are not in a perfect tetrahedral environment but rather in distorted tetrahedra with *C*_s_ local symmetry, such that the 1E splits into nondegenerate 1A′ and 1A″ states.^28,69,70^ There are also two Mn–O vibronic sidebands of lower intensity at ∼1225 and 1280 nm, assigned to the O–Mn–O bending and Mn–O stretching vibrations, respectively, as documented in the literature.^37,70,71^ A broad band of much lower intensity is just discernible around 1025 nm, due to emission from the higher-lying ^3^T_2_ state. Its low intensity reflects the low thermally activated population of this state at room temperature. The corresponding spectra for Ba_5_(V_0.97_O_4_)_3_Cl:Mn_0.03_ are shown in Figure S9b.

The absorption spectrum, obtained by applying the Kubelka–Munk function to diffuse reflectance data (Figure 4a), shows a broad, intense absorption band due to the charge-transfer transition from the p-orbitals of the oxide ions to vacant d-orbitals of the V^5+^ ions. The inset shows the Mn^5+^ bands that arise from the ^3^T_1_(F) ← ^3^A_2_, ^3^T_1_(P) ← 3A_2_, and ^3^T_2_ ← 3A_2_ transitions, alongside the sharper peaks of the spin-forbidden 1A_1_ ← 3A_2_ and 1E ← 3A_2_ transitions. The corresponding spectra for Ba_5_(V_0.97_O_4_)_3_Cl:Mn_0.03_ are shown in Figure S8a.

The quantum yields determined for Sr_5_(V_0.97_O_4_)_3_Cl:Mn_0.03_ and its Ba^2+^ analogue are 47(2) and 51(2)%, respectively. High brightness is important, especially for potential biomedical applications such as luminescence thermometers, to complement efficient NIR excitation and emission through tissue and skin.^72^ These quantum yields are remarkable and rare for NIR emitters, with most examples in the literature exhibiting values <20%. Recently, Ca_6_Ba­(PO_4_)_4_O:0.5% Mn^5+^ was found to have a quantum yield of 38(2)%.^34^ Ba_3_(PO_4_)_2_-based Mn^5+^-doped materials show quantum yields up to ∼40%,^28,73^ while values >20% were reported for analogous vanadates, with a maximum of 51% for Ba_3_(VO_4_)_2_:1.0% Mn^5+^.^35^ In all these cases, the samples were shown to be phase-pure by PXRD. Exceptionally high quantum efficiency (82%) was recently reported for Ca_6_Ba­(PO_4_)_4_O: 0.003Mn^5+^/0.003 Bi^3+^; however, a close inspection of the raw PXRD patterns and the Rietveld fits shows the presence of unidentified peaks, indicating that the sample on which the physical property measurements were carried out was not pure.^74^

### 2.4 Luminescence Thermometry

Sr_5_(V_0.97_O_4_)_3_Cl:Mn_0.03_ and Ba_5_(V_0.97_O_4_)_3_Cl:Mn_0.03_ were selected for detailed variable-temperature luminescence measurements based on the initial screening of room-temperature luminescence performed on all samples (Figure S8).

#### 2.4.1 LIR Luminescence Thermometry

Sr_5_(V_0.97_O_4_)_3_Cl:Mn_0.03_ was initially investigated for luminescence thermometry at physiologically relevant temperatures by LIR, as shown in Figure 5 (note that a logarithmic intensity scale is used in Figure 5a). The main 1E narrow emission peak intensity decreases relative to the weaker, broader ^3^T_2_ peak with increasing temperature, due to thermal population of the latter state from the former. This thermalisation is key to Boltzmann-type LIR thermometry, as shown by the equation:

LIR=IHIL=IT32I1E=B×exp(−ΔEkBT) 2

where *I*_H_ and *I*_L_ are the emission intensities for the higher and lower energy ^3^T_2_ and ^1^E states, respectively, Δ*E* is the energy difference between these two states, *B* is a frequency constant, *k*_B_ is the Boltzmann constant, and *T* is the absolute temperature.

**Figure 5:**
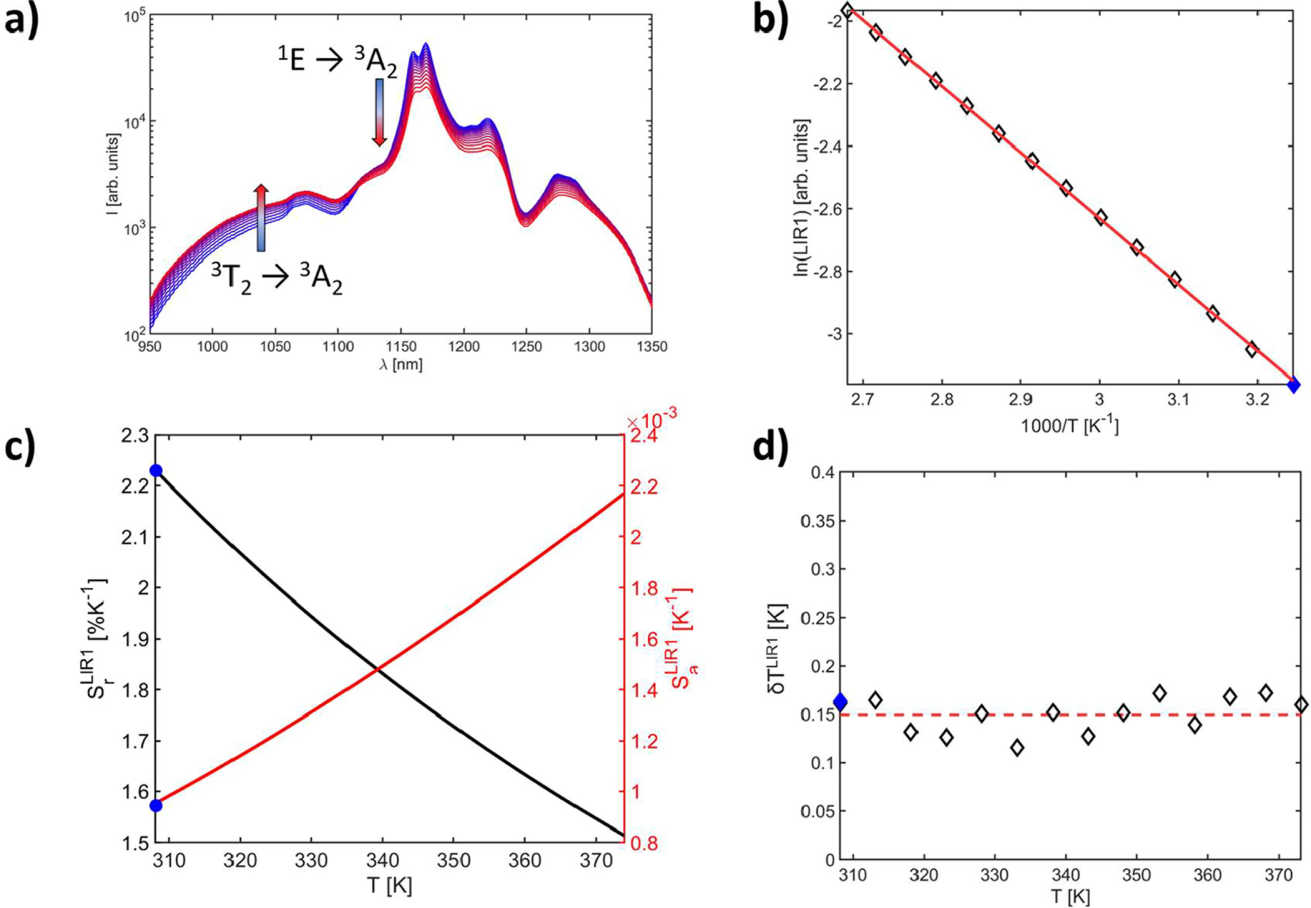
Normalized variable-temperature luminescence data for Sr_5_(V_0.97_O_4_)_3_Cl:Mn_0.03_. (a) Logarithmic-scaled VT emission intensity showing the changes in intensity of the ^3^T_2_ and 1E bands as temperature increases from 35 to 100 °C; (b) linearized Boltzmann equation, fitting the LIR data (diamond markers; average of 75 scans); (c) plot of the experimentally obtained sensitivities; relative (black, left axis) and absolute (red, right axis); (d) temperature resolution of each experimental measurement—the filled blue diamond represents 308 K.

At each temperature, 75 readings were recorded and averaged to give LIR values. To obtain these values for Sr_5_(V_0.97_O_4_)_3_Cl:Mn_0.03_, the ^3^T_2_ band intensity was integrated between 950 and 1050 nm and the ^1^E peak between 1140 and 1200 nm. Using eq 6, the energy gap between the two thermally coupled states was determined to be 1471 cm^–1^ for Sr_5_(V_0.97_O_4_)_3_Cl:Mn_0.03_ and 1360 cm^–1^ for Ba_5_(V_0.97_O_4_)_3_Cl:Mn_0.03_ (Figures 5b and S10b). The temperature-invariant constant, *B*, was calculated to be 41.21 for Sr_5_(V_0.97_O4)_3_Cl:Mn_0.03_. The LIR values were fitted using the Boltzmann LIR equation (red line in Figures 5b and S10b). From the Sr_5_(V_0.97_O_4_)_3_Cl:Mn_0.03_ energy difference of 1471 cm^–1^, the absolute and relative sensitivities of the thermometer (*S*_a_, and *S*_r_ respectively) were calculated from the following two equations:

$$S_{a}=\left|\frac{\Delta \mathrm{LIR}}{\Delta T}\right|=\frac{\Delta E}{k_{B}T^{2}}B\exp\left(-\frac{\Delta E}{k_{B}T}\right)$$ 3

$$S_{r}=\left|\frac{1}{\Delta \mathrm{LIR}}\frac{\Delta \mathrm{LIR}}{\Delta T}\right|\times 100=\frac{\Delta E}{k_{B}T^{2}}\times 100$$ 4

The maximum *S*_a_ value for Sr_5_(V_0.97_O_4_)_3_Cl:Mn_0.03_ obtained at 373 K is 2.2 × 10^–3^ K (Figure 5c, red curve). In terms of *S*_r_, which allows for comparison between other readout methods, the best performance observed for Sr_5_(V_0.97_O_4_)_3_Cl: Mn_0.03_ is 2.23% K^–1^ at 308 K (Figure 5c, black curve).

The temperature resolution, δ*T*, which is the smallest change in temperature which can effect a perceivable change to the LIR readout, was calculated using the following equation:

$$\delta T=\frac{\sigma_{r}}{S_{r}}$$ 5

where σ_r_ is the relative uncertainty estimated from 75 consecutive measurements at a given temperature. As shown in Figure 5d, the average temperature resolution for Sr_5_(V_0.97_O_4_)_3_Cl:Mn_0.03_ across the temperature range investigated is 0.15 K (indicated by the red dashed lines.) At physiological temperature (35 °C, 308 K), the temperature resolution is 0.16 K. Corresponding data for Ba_5_(VO_4_)_3_Cl:Mn_0.03_ are given in Figure S10 and Table S6 (the latter summarizes the comparison of the two materials). These temperature sensitivity and resolution parameters reflect excellent LIR thermometry performance of both materials compared to NIR BW-2 luminescence thermometers in the literature, superior to Ca_6_Ba­(PO_4_)_4_O:0.5% Mn^5+^ (which has LIR *S*_r_ of 2.04% K^–1^ at 293 K with an average temperature resolution of 0.21 K (Table S7).^34^

#### 2.4.2 Band-Shift and Bandwidth (fwhm) Luminescence Thermometry

Band-shift luminescence thermometry is based on the change in the position of the maximum intensity of the peak centroid. In this work, the position of the highest intensity emission peak was determined at each temperature and converted to wavenumber (cm^–1^). The experimental band-shift data for Sr_5_(V_0.97_O_4_)_3_Cl:Mn_0.03_ were fitted using a second order polynomial:

$$E_{\max}^{\mathrm{Av}}(T)=aT^{2}+bT+c$$ 6

The parameters obtained from fitting the band-shift data for Sr_5_(V_0.97_O_4_)_3_Cl:Mn_0.03_ are *a* = 3.574 × 10^–4^ K^–2^ cm^–1^, *b* = −3.004 × 10^–1^ K^–1^ cm^–1^, *c* = 8605.624 cm^–1^ (Table S3, and those calculated for Ba_5_(V_0.97_O_4_)_3_Cl:Mn_0.03_ are given in Table S4).

Figure 6a gives the fit obtained for Sr_5_(V_0.97_O_4_)_3_Cl:Mn_0.03_ and shows the red-shifting of the 1E peak maximum energy from 8547 to 8543 cm^–1^ as temperature increases from 308 to 373 K. Sr_5_(V_0.97_O_4_)_3_Cl:Mn_0.03_ demonstrates a maximum *S*_a_ of 0.08 cm^–1^ K^–1^ at 308 K (Figure 6b). The average temperature resolution, indicated by the dashed red line in Figure 6c, is ∼0.2 K, with a temperature resolution of ∼0.05 at 308 K. Corresponding data for the Ba-analogue are given in Figure S11 and Table S6.

**Figure 6:**
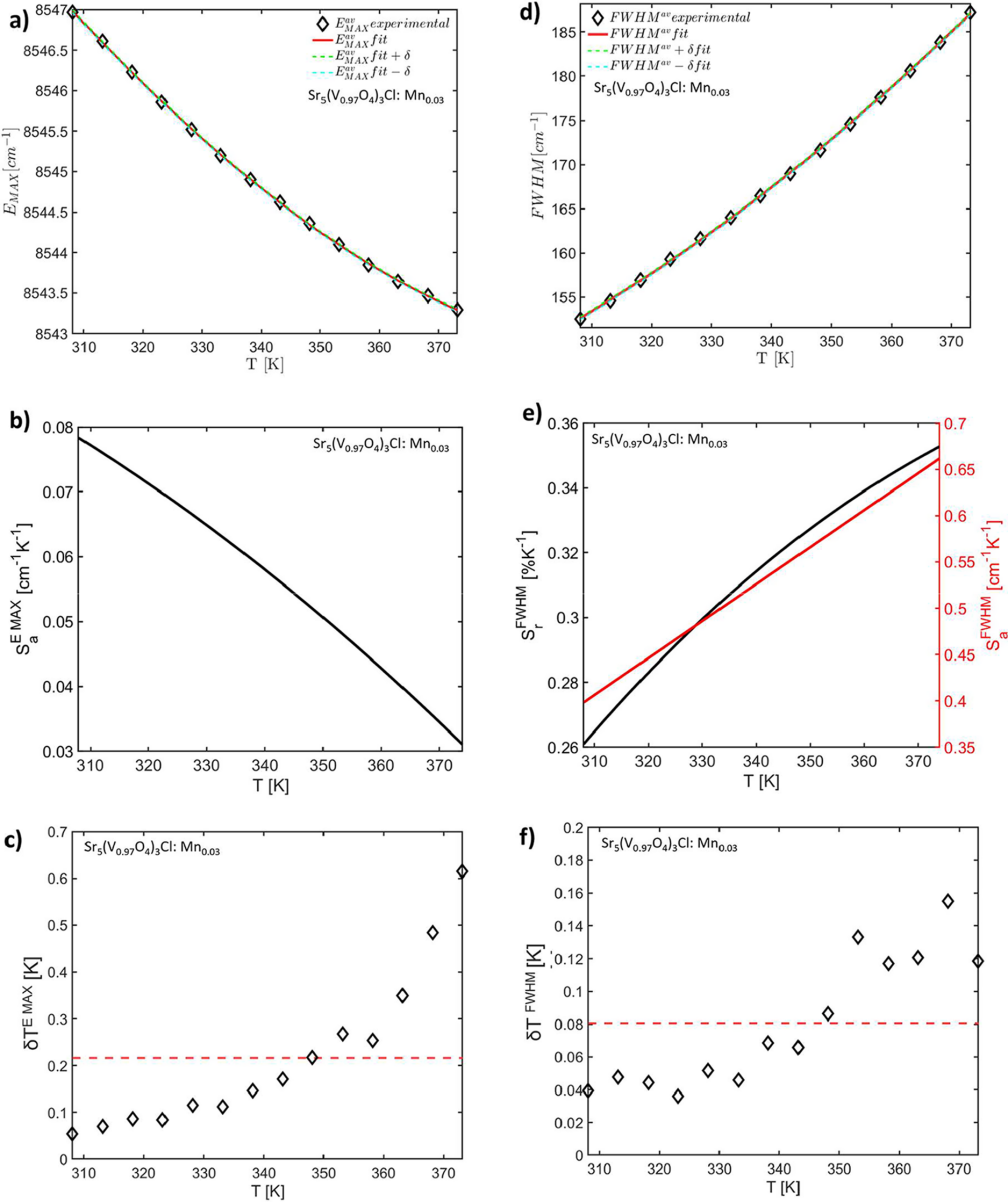
Band-shift thermometry data analysis for Sr_5_(V_0.97_O_4_)_3_Cl:Mn_0.03_: (a) Fitted peak maxima data as absolute energy (cm^–1^) with dashed lines showing a 95% confidence interval; (b) absolute sensitivity, *S*_a_; (c) temperature resolution (red dashed line shows the average over the temperature range observed). Bandwidth thermometry data analysis: (d) fitted peak width data as absolute energy (cm^–1^) with dashed lines showing a 95% confidence interval; (e) absolute sensitivity, *S*_a_ (red) and relative sensitivity, *S*_r_ (black). (f) Temperature resolution (red dashed line shows the average over the temperature range observed).

Bandwidth thermometry measures the change in width (fwhm in units of cm^–1^) at half the maximum intensity of a selected peak as a function of temperature. For Sr_5_(V_0.97_O_4_)_3_Cl:Mn_0.03_, the fwhm experimental data were fitted using a second-order polynomial:

$$\mathrm{FWHM}(T)=iT^{2}+jT+k$$ 7

and the parameters obtained were *i* = 1.996 × 10^–3^ K^–2^ cm^–1^, *j* = −8.316 × 10^–1^ K^–1^ cm^–1^, and *k* = 219.342 cm^–1^; corresponding parameters for the Ba-analogue are listed in Table S5. Figure 6d shows the fitted fwhm data, indicating a broadening of the 1E peak of Sr_5_(V_0.97_O_4_)_3_Cl:Mn_0.03_ from 153 to 187 cm^–1^ as temperature increases from 308 to 373 K. Sr_5_(V_0.97_O_4_)_3_Cl:Mn_0.03_ has a maximum *S*_a_ of 0.66 cm^–1^ K^–1^ at 373 K (Figure 6e) and a maximum *S*_r_ of ∼0.35% K^–1^, with a *S*_r_ of 0.26% K^–1^ at 308 K. The average δ*T* is ∼0.08 K (dashed red line in Figure 6f), with a temperature resolution of ∼0.04 at 308 K. The performance of Ba_5_(V_0.97_O_4_)_3_Cl:Mn_0.03_ is comparable (Figure S10, Table S6).

#### 2.4.3 Multiparametric Luminescence Thermometry

The comparison of the performance between three single-parameter thermometry methods and the multiparametric method (described in detail in the SI) for Sr_5_(V_0.97_O_4_)_3_Cl:Mn_0.03_ was performed experimentally by acquiring 75 spectroscopic measurements at each temperature. Values of the key coefficients obtained by multilinear parametric (MLR) analysis are presented in Table 1.

**Table 1:** Constant Term, β_0_, and Slope Coefficients, β_*i*_, Obtained by MLR Applied to the Sr_5_(V_0.97_O_4_)_3_Cl:Mn_0.03_ Dataset

β_0_ [K]	β_1_ [%]	β_2_ [%]	β_3_ [%]
–0.050	11.03	30.19	58.80

The maximum relative sensitivity is 3.14% K^–1^ at 308 K (Figure 7a), with only a very small decrease with increasing temperature (to 2.59% K^–1^ at 373 K). Similarly, the accuracy (Figure 7b) and precision (the standard deviation of repeated temperature measurements, Figure 7c) for each method and temperature are calculated. Figure 7d–g show the distributions of calculated temperatures at the nominal temperature of 313 K, for LIR, band-shift, and fwhm-based single-parameter methods (Figure 7d,e), as well as for the multiparametric method that utilizes all three thermometric indicators (Figure 7g). Based on these data and considering the entire temperature range investigated, the temperature determined by MLR (*T*_MLR_) has the best accuracy, while retaining the precision of the most precise single-parameter method (in this case, the fwhm one).

**Figure 7:**
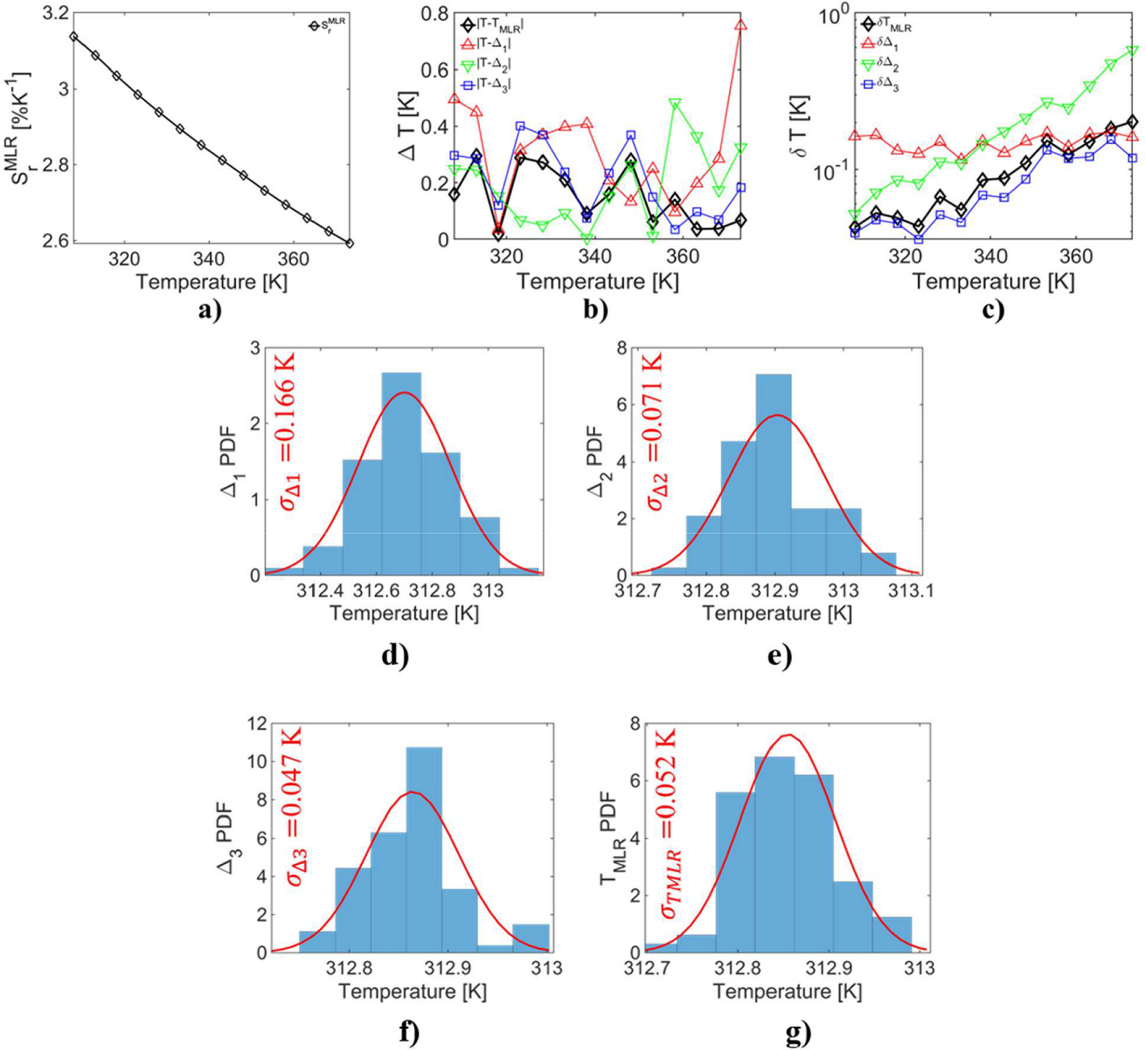
(a) Relative sensitivity of MLR, (b) accuracy (ΔT) and (c) the precision (δ*T*) of single-parametric (LIR: red △ symbol and line; ^1^E peak maximum energy: green ▽ triangle symbol and line; and ^1^E peak fwhm: blue □ symbol and line) and multiparametric temperature (black ◊ symbol and line) readings from the NIR emission of Sr_5_(V_0.97_O_4_)_3_Cl:Mn_0.03_ at different temperatures; (d–g) distributions of temperatures measured by single-parametric (LIR, ^1^E peak maximum, and ^1^E peak fwhm) and multiparametric thermometry, respectively (nominal temperature 313.15 K); temperature distributions were fitted to the normal distribution (red line), and the standard deviations are written on each graph.

Tables S7 and S8 summarize the best-performing Mn^5+^-containing thermometers reported in the literature, and selected luminescence thermometers that emit within at least one of the three biological windows of transparency. Table S7 shows that, to our knowledge, Sr_5_(V_0.97_O_4_)_3_Cl:Mn_0.03_ exhibits *S*_r_ values among the highest reported for Mn^5+^-activated thermometry phosphors, with a maximum *S*_r_ of 2.23% K^–1^ (308 K) for the LIR readout, and an even higher *S*_r_ of 3.14% K^–1^ (308 K) for the MLR readout. Two Mn^5+^-containing Ca_6_BaP_4_O_17_ phosphors have *S*_r_ of similar values of 2.00% K^–1^ (298 K) and 2.05% K^–1^ (293 K) for LIR readout, with temperature resolution of 0.2 K. Relative sensitivity *S*_r_ of 2.91% K^–1^ (300 K) for LIR readout has recently been reported for Sr_5_(PO_4_)_3_F, although the temperature resolution was not given in that case.^75^

#### 2.4.4 Parameter-Free Full-Spectrum-Based Luminescence Thermometry

The variable-temperature luminescence spectra collected on Sr_5_(V_0.97_O_4_)_3_Cl:Mn_0.03_ were analyzed further using parameter-free full-spectrum-based methods (described in detail in the SI). For both Principal Component Analysis (PCA) and Support Vector Regression (SVR) methods, 35 randomly selected spectra at each of 14 stabilized temperatures were used for training (Figure 8a), while the remaining 40 were used to test the thermometric capabilities of the luminescence thermometer. Testing was performed by forming a distribution of temperature readouts at each stabilized temperature, allowing direct measurement of accuracy Δ*T* (the absolute difference between the mean of the temperature readout distribution and the stabilized temperature) and resolution d*T* (the standard deviation of the temperature readout distribution at each stabilized temperature).

**Figure 8:**
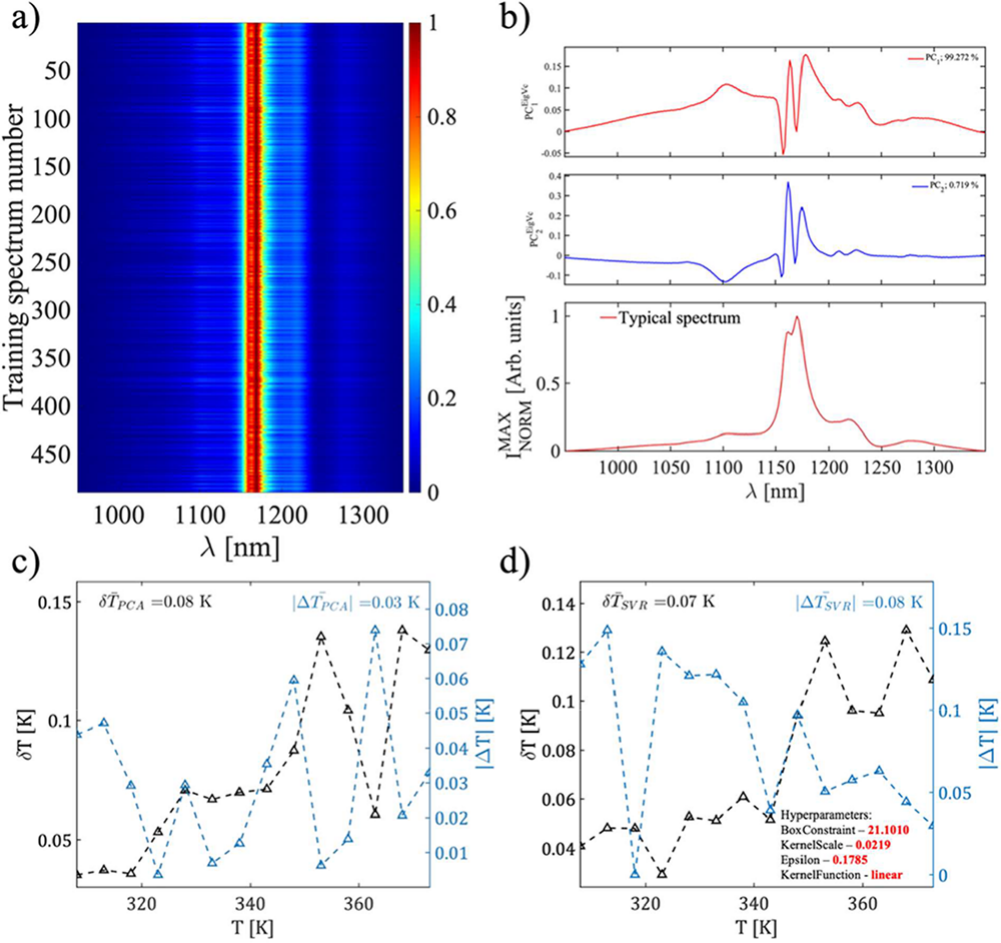
(a) Training data set consisting of 490 randomly shuffled spectra (35 spectra at each of 14 stabilized temperatures); (b) typical spectrum (bottom) and first two principal components loading vectors with the total variance contribution capture by each given; (c) results of PCA and (d) the accuracy (Δ*T*) and resolution (δ*T*) of the SVR methods when applied to the test spectra, with average values over the entire temperature range investigated, and SVR-optimized hyperparameters given in the inset to (d).

In PCA, often just the first principal component constitutes a low-dimensional, noise-filtered thermometric fingerprint of each spectrum, derived entirely from the data structure itself without any free parameters governing feature extraction (Figure 8b). Figure 8c presents the resulting accuracy (Δ*T*) and resolution (δ*T*) values as a function of temperature, obtained by projecting the test spectra of Sr_5_(V_0.97_O_4_)_3_Cl:Mn_0.03_ onto the first principal component (PC1) loading vector only. PC1 encapsulates more than 99% of the total spectral variance and, using the Euclidean metric distance, maps the projections to temperature by interpolating between calibration-set PC1 values (values obtained during the training phase at stabilized temperatures). This approach yielded average accuracy ( $\Delta \overset{-}{T}$ ) and resolution ( $\delta \overset{-}{T}$ ) values of 0.03 and 0.08 K, respectively.

In the SVR approach, the MATLAB *fitrsvm* function was governed by a small number of hyperparameters: the choice of kernel function and kernel scale (Figure 8d, KernelFunction and KernelScale), the regularisation constant (Figure 8d, BoxConstraint), and the tolerance margin (Figure 8d, Epsilon). These hyperparameters control the learning process rather than encoding any assumption about spectral physics, leaving the thermometric information extraction entirely data-driven. The resulting accuracy (Δ*T*) and resolution (δ*T*) values obtained for Sr_5_(V_0.97_O_4_)_3_Cl:Mn_0.03_, together with their averages of 0.08 and 0.07 K across the full thermometric domain, are shown in Figure 8d.

In the GPR approach, variable-temperature Sr_5_(V_0.97_O_4_)_3_Cl:Mn_0.03_ emission spectra were accepted as input vectors, returning a temperature estimate accompanied by a confidence interval (Figure 9a) that reflects not only what temperature it predicts, but how much that prediction should be trusted (MATLAB *fitrgp* and *predict* functions were used). This method gives accuracy better than Δ*T* < 0.1 K over the investigated temperature range and resolution lower than δ*T* < 0.14 K, with average values of 0.05 and 0.07 K, respectively.

**Figure 9:**
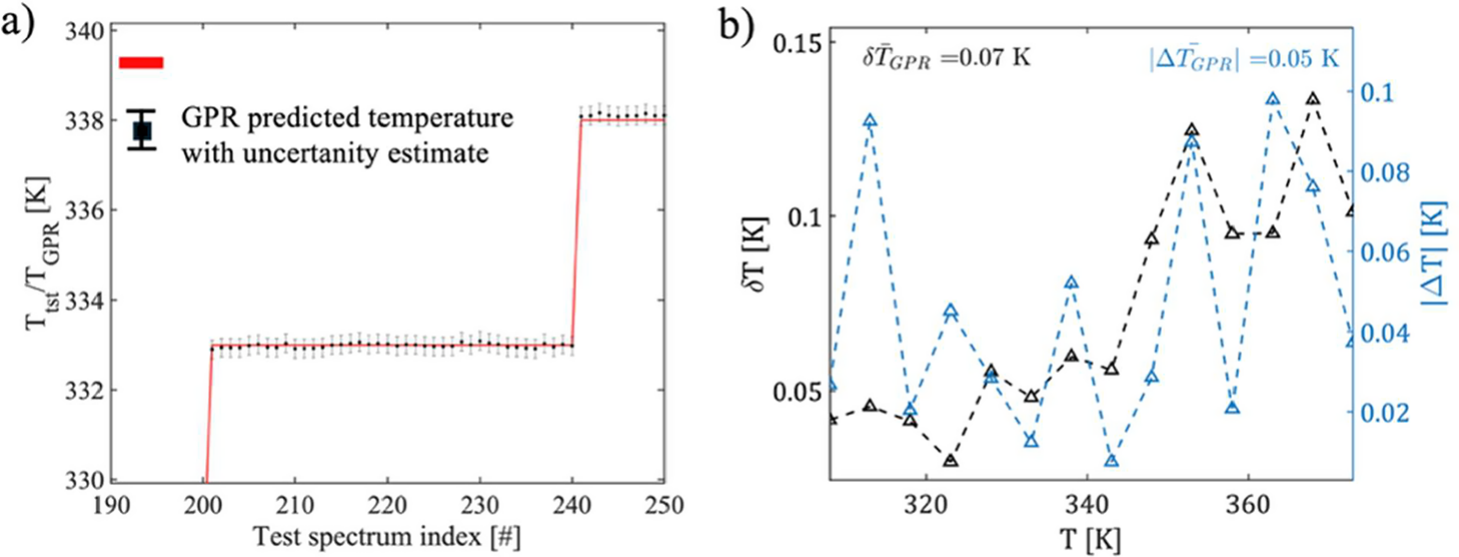
(a) Part of the temperature predictions with uncertainties (black square markers with bars) obtained by the GPR algorithm, based on testing spectra recorded at stabilized temperature (red line); (b) results of GPR method accuracy (Δ*T*) and resolution (δ*T*) when applied to the test spectra, with average values over the entire temperature range investigated.

## 3 Conclusions

Following the initial screening of a series of A_5_(V_1–*x*_O_4_)_3_Cl:Mn_*x*_ materials (A = Ca^2+^, Sr^2+^, Ba^2+^; *x* = 0.00, 0.01, 0.03, 0.05) and their characterization by X-ray diffraction, solid-state NMR and room temperature luminescence measurements, two materials obtained as pure samples by sol–gel synthesis, Sr_5_(V_0.97_O_4_)_3_Cl:Mn_0.03_ and Ba_5_(V_0.97_O_4_)_3_Cl:Mn_0.03_, were evaluated as luminescence thermometers. Three single-parametric luminescence thermometry methods were explored for both materials, giving high sensitivity performance indicators at physiological temperature: *S*_r_ = 2.23% K^–1^ (LIR), *S*_a_ = 0.08 cm^–1^ K^–1^ (band-shift), *S*_r_ = 0.26% K^–1^ (bandwidth) for Sr_5_(V_0.97_O_4_)_3_Cl:Mn_0.03_; and *S*_r_ = 2.06% K^–1^ (LIR), *S*_a_ = 0.10 cm^–1^ K^–1^ (band-shift), *S*_r_ = 0.31% K^–1^ (bandwidth) for Ba_5_(V_0.97_O_4_)_3_Cl:Mn_0.03_. Crucially, this is accompanied by high temperature resolution, particularly for the Sr-containing material, with the values of 0.16 (LIR), 0.05 (band-shift), and 0.04 K (bandwidth). On this basis, the multiparametric thermometry potential of Sr_5_(V_0.97_O_4_)_3_Cl:Mn_0.03_ was investigated, and MLR analysis gave remarkable performance key parameters at 308 K: temperature relative sensitivity *S*_r_ = 3.14% K^–1^ and temperature resolution of 0.042 K. The multiparametric thermometry approach gave even better accuracy than the best individual readout (LIR), while retaining the exceptionally good temperature determination precision of the best single-parametric method (fwhm). Finally, we demonstrated that all full-spectrum methods dramatically improved temperature readout accuracy, without *a priori* identification of temperature-dependent features, yielding average values ranging from 0.03 (PCA) to 0.08 K (SVM), while simultaneously maintaining average resolution values of 0.08 (PCA) and 0.07 K (SVM and GPR) across the entire temperature range.

Coupled with the emission lying in BW-2, the remarkable brightness (QY 47%), and the thermal stability at both crystallographic long-range and short-range local structure length scales demonstrated by variable-temperature X-ray diffraction and solid-state NMR, respectively, the remarkable thermometry performance indicators demonstrated here make Sr_5_(V_0.97_O_4_)_3_Cl:Mn_0.03_ a very promising candidate for further development aimed at applications in luminescence thermometry at physiological temperatures.

## 4 Experimental Section

### 4.1 Synthesis

A series of polycrystalline materials with the general formula A_5_(V_1–*x*_O_4_)_3_Cl:Mn_*x*_, where A = Ca^2+^, Sr^2+^, Ba^2+^ and *x* = 0.00, 0.01, 0.03, 0.05, were initially synthesized by a conventional solid-state method. Stoichiometric amounts of CaCO_3_ (Sigma-Aldrich, ≥99%), BaCO_3_ (Sigma-Aldrich, 99%), SrCO_3_ (Sigma-Aldrich, >99.9%), NH_4_VO_4_ (Sigma-Aldrich, > 99%), NH_4_Cl (Sigma-Aldrich, >99.5%), and Mn_2_O_3_ (Sigma-Aldrich, 99%) were weighed and ground using an agate mortar and pestle. The mixtures were heated in alumina crucibles at temperatures between 600 and 1100 °C for 3 to 72 h with intermittent grinding; synthesis details for specific compositions are given in Table S1. A slower heating rate of 1 °C/min was used for the first firing due to the initial release of gases from reactants, and a faster heating rate of 5 °C/min was used for further heating. Powder X-ray diffraction (PXRD) consistently showed the presence of impurities, suggesting some Cl^–^ loss in these samples.

Further samples of Sr_5_(V_0.97_O_4_)_3_Cl:Mn_0.03_ and Ba_5_(V_0.97_O_4_)_3_Cl:Mn_0.03_ were therefore synthesized by the sol–gel method and using an excess of chloride ions.^76^ Sr­(NO_3_)_2_ (Sigma-Aldrich, ≥98%) or Ba­(NO_3_)_2_ (Sigma-Aldrich, 99.999%), NH_4_VO_4_ (Sigma-Aldrich, ≥99%), and Mn_2_O_3_ (Aldrich, 99%) were weighed stoichiometrically; NH_4_Cl (Alfa Aesar, 99.999%) was weighed in excess of 6–10 mol %. Citric acid (Sigma-Aldrich, >99.5%) was weighed out in a 2:1 ratio relative to the metal nitrate. The Sr­(NO_3_)_2_, NH_4_VO_4_, NH_4_Cl, and Mn_2_O_3_ were added to 100 mL of deionized water with heating and stirring. Citric acid was then added to form a transparent yellow solution. NH_3(aq)_ (Fischer-Sci, 35%) was added dropwise until the solution pH reached 7. The solution was heated with stirring until a viscous blue gel formed. The gel was left to stand for 48 h before being placed into a muffle furnace for a precalcination in an alumina crucible at 250 °C for 2 h (0.1 °C/min heating rate). The product was ground, pressed into 10 mm (o.d.) pellets via a pneumatic pellet press (5 tonnes), and heated at 750 °C in 12 h intervals (1.0 °C/min heating rate) until phase purity was confirmed by PXRD. Homogenous distribution of the Mn^5+^ activator ions was confirmed using chemical mapping via EDX using a JEOL 2100F FEG transmission electron microscope (TEM) with a single tilt holder. Sample powder was placed into a glass vial with isopropanol, homogenized using an ultrasound bath (10 min cycle), and a single drop of suspended sample was placed onto a lacey carbon film 200 copper mesh disk (Agar scientific). TEM images were captured via a Gatan Orius CCD camera. Chemical mapping was done using an Oxford INCAx-sight Si­(Li) detector for EDX, and software Aztec 5.1.

### 4.2 Powder X-ray Diffraction

PXRD data were collected on a Bruker AXS D8 Advance diffractometer using CuK_α_ radiation, a Lynx-Eye detector, and a HTK1200 furnace. Routine diffraction patterns were recorded in the range of 10° < 2θ < 90°, with a step size of 0.02° and a step time of 2.5 s/step. Variable-temperature data were recorded in the range of 10° < 2θ < 90° with a step size of 0.02° and a step time of 0.5 s/step. All data were analyzed by the Rietveld method implemented in TOPAS Academic v7 software.^77,78^ When PXRD patterns were analyzed for routine phase identification purposes, the refined parameters included unit cell parameters, an overall isotropic atomic displacement parameter, scale factor, sample height displacement, pseudo-Voigt peak shape function terms, and background polynomial terms. For detailed structural analysis of Sr_5_(V_0.97_O_4_)_3_Cl:Mn_0.03_ and Ba_5_(V_0.97_O_4_)_3_Cl:Mn_0.03_, PXRD patterns were collected in the range of 10° < 2θ < 120° with a step size of 0.02° and a step time of 6.5 s/step. Refined parameters included the sample height displacement, background polynomial terms, pseudo-Voigt peak-shape function terms, unit cell parameters, atomic fractional coordinates (with bond valence sum restraints on V atoms), and individual isotropic atomic displacement parameters.

### 4.3 Solid-State Nuclear Magnetic Resonance

^51^V solid-state NMR (ssNMR) magic angle spinning measurements were conducted on samples of ∼0.2 g contained within a 4 mm (o.d.) probe using a Bruker Advance III HD spectrometer. Spectra were acquired at a spin rate of 12 kHz. All direct-excitation ^51^V spectra were acquired with a 1 μs 30-degree solid pulse. The spectra were acquired with a recycle delay of 0.4 s determined on the sample. Spectra were referenced with neat tetramethylsilane on ^1^H and adjusted the frequency using the method described by Harris et al.^79^ Variable-temperature ssNMR data were collected between 20 and 100 °C in 10 °C increments by 30 min scans. Pb­(NO_3_)_2_ was used as a temperature reference.

### 4.4 Photoluminescence Spectroscopy

Room-temperature emission measurements were performed using a Horiba Jobin-Yvon Fluorolog-3 spectrometer with a xenon lamp excitation source; emitted light in the NIR was detected with a thermoelectrically cooled NIR photomultiplier tube (Hamamatsu H10330B). Samples were contained within quartz capillaries (3 mm o.d.) held in the sample chamber within a 3D-printed holder. Long-pass filters (400 and 850 nm) were used to eliminate scattered excitation harmonics (λ_em_/2 and 2λ_ex_) from excitation and emission spectra, respectively. Diffuse reflectance spectroscopy (DRS) measurements were undertaken using a Shimadzu UV-2600 spectrophotometer (Shimadzu corporation, Tokyo, Japan) with an integrating sphere, using BaSO_4_ as an optically inert reference over the 220–1350 nm range. DRS data were transformed to absorption intensity data using the Kubelka–Munk function, *F*(*R*) = *k*/*s* = (1 – *R*)^2^/2*R*, where *R* is the reflectance. Variable-temperature emission spectra were recorded on 5 mm pellets between 308 and 373 K, using an Ocean Insight FX NIR-Quest spectrometer with bifurcated fiber-optic cable and a custom-built heating stage.^80^ The excitation source was an Ocean Insight LSM-365A LED at 365 nm. The quantum yield (QY) was measured using an Ocean Insight ISS-REF integrating sphere upon excitation at 635 nm with the same LED source, in conjunction with Ocean Insight OCEAN-FX-XR1-ES and NIR Quest+ spectrometers. Ocean Insight WS-1 Diffuse Reflectance Standard was used as the reference.

### 4.5 Multiparametric Luminescence Thermometry

It is possible to combine the luminescent thermometry methods of LIR, band shift, and bandwidth by using multiparametric linear regression (MLR)-a statistical method that uses two or more explanatory variables to determine the outcome of a response variable. In simple terms, MLR is a multidimensional extension of least-squares regression.

Here, values of LIR, *E*_max_, and fwhm are the desirable independent explanatory variables, and temperature is the response variable. Unfortunately, the prerequisite for using MLR is that there be a linear relationship between the dependent variables and the independent variables and that the independent variables are not too highly correlated with each other. While LIR, *E*_max_, and fwhm are indeed not highly correlated with each other, none of them has a linear relationship with *T* in the observed temperature range. There have been successful applications of MLR demonstrated over the limited temperature range wherein approximate linearity can be assumed.^81,82^ In addition, although it is not strictly required, the values of independent variables should be of the same order of magnitude, due to the nature of fitting procedures that favor variables with larger values.

To overcome the nonlinearity and the magnitude problem, here we can introduce the thermometric parameters Δ_*i*_ (independent variables) that are simply temperature readouts obtained from single-parameter readouts:^36^

$$\Delta_{1}=T_{\mathrm{LIR}}=\frac{\Delta E}{k(\log(B)-\log(\mathrm{LIR}))}$$ 8

$$\Delta_{2}=T_{E_{\max}}=\frac{-b-\sqrt{b^{2}-4a(c-E_{\max})}}{2a}$$ 9

$$\Delta_{3}=T_{\mathrm{FWHM}}=\frac{-j-\sqrt{j-4i(k-\mathrm{FWHM})}}{2i}$$ 10

Parameters Δ*E*, *B*, *a*, *b*, *c*, *i*, *j,* and *k* are obtained by fitting experimental results to the above-mentioned models (Sections 2.4 and 2.5). Using multiple linear regression, one can represent the linear relationship between these parameters and the thermometric temperature (the dependent variable) such that

$$T_{\mathrm{MLR}}=\beta_{0}+\beta_{1}\Delta_{1}+\beta_{2}\Delta_{2}+\beta_{3}\Delta_{3}$$ 11

where β_0_ is the constant term and β_1_, β_2_, and β_3_ are the slope coefficients for each of the three explanatory variables. One should notice that in this representation, β_1_, β_2_, and β_3_ have a role of significance coefficients for each of the three temperature readouts Δ_*1*_, Δ_2_, and Δ_3_, respectively (β_1_ + β_2_ + β_3_ ≈ 1). Note also that in this specific case, β_0_ is a measure of accuracy correction since, if all Δ_*i*_ parameters show the same temperature readout, then β_0_ represents the *T*_MLR_ offset from that value. Based on these β values, the relative error of the MLR method can be calculated as^82^

$$S_{r}^{\mathrm{MLR}}[\%K^{-1}]=100\%\sqrt{\frac{1}{(\beta_{1}\Delta_{1}{)}^{2}}+\frac{1}{(\beta_{2}\Delta_{2}{)}^{2}}+\frac{1}{(\beta_{3}\Delta_{3}{)}^{2}}}$$ 12

### 4.6 Parameter-Free Full-Spectrum-Based Luminescence Thermometry

Conventional thermometric approaches require manual identification and isolation of specific spectral parameters, intensity ratios between chosen bands, peak centroids, or spectral widths, decisions that embed assumptions about which parts of the spectrum carry thermal information, and which do not.

Principal component analysis (PCA)^83^ and support vector regression (SVR)^84^ make no such assumptions and offer a parameter-free entry point into full-spectrum luminescence thermometry that sidesteps one of the field’s most persistent challenges: physics-informed selection of spectral features. Rather than requiring the user to define which spectral observables to feed into a calibration model, both methods accept the entire emission spectrum as their input. The difference between PCA and SVR lies in the way the mapping to readout temperature is performed—unsupervised in the case of PCA (temperature data are not used in the training process, with spectra mapped to principal components and temperature assigned a posteriori) and supervised in the SVR case (temperature data labeled and used during the training process). Applied to a training data set of emission spectra recorded across a temperature range, PCA automatically discovers the directions of maximum spectral variance and projects the full, high-dimensional spectral space onto a compact set of principal components. When temperature is the dominant experimental variable, the leading principal components naturally capture thermally driven spectral evolution in its entirety—not merely the variance visible in a pair of selected bands, but the collective, correlated response of the whole emission profile. SVR approaches the same full-spectrum thermometric problem from the supervised direction but with an equally parameter-free philosophy. SVR accepts the entire emission spectrum as its input vector and autonomously learns the nonlinear mapping from spectral shape to temperature during training on temperature-labeled measurements, a defining characteristic of supervised learning methods. The kernel allows SVR to operate implicitly in a high-dimensional feature space constructed from the raw spectral data, identifying the combination of spectral variations that best predicts temperature without any explicit decomposition or band selection by the researcher.

Gaussian Process Regression (GPR) is a nonparametric, probabilistic supervised learning method that models the relationship between inputs and outputs by placing a prior distribution directly over functions rather than over a fixed set of parameters.^85^ At its core, a Gaussian process is fully specified by a mean function and a covariance—or kernel—function, the latter encoding prior assumptions about the smoothness and length scale of the underlying functional relationship. When trained on labeled data, GPR updates this prior into a posterior distribution over functions consistent with the observations, yielding not only a point prediction for each new input but a full probability distribution—and therefore a natural, mathematically rigorous uncertainty estimate, at every predicted value. This built-in predictive uncertainty, absent in SVR and only indirectly accessible through PCA-based interpolation, is arguably GPR’s most distinctive practical asset.^86^

## Supplementary Material



## Notes

Synthetic conditions used for all samples; Rietveld fits against powder X-ray diffraction data for all samples prepared by the solid-state route; the final Rietveld fit for Ba_5_(V_0.97_O_4_)_3_Cl:Mn_0.03_ synthesized by the sol–gel route; structural parameters for Sr_5_(V_0.97_O_4_)_3_Cl:Mn_0.03_ and Ba_5_(V_0.97_O_4_)_3_Cl:Mn_0.03_ obtained from Rietveld refinements; EDX chemical maps for Sr_5_(V_0.97_O_4_)_3_Cl:Mn_0.03_, and Ba_5_(V_0.97_O_4_)_3_Cl:Mn_0.03_; ^51^V solid state NMR spectra for selected samples; absorption and emission spectra and luminescence thermometry data for Ba_5_(V_0.97_O_4_)_3_Cl:Mn_0.03_; thermometric figures of merit for Sr_5_(V_0.97_O_4_)_3_Cl:Mn_0.03_ and Ba_5_(V_0.97_O_4_)_3_Cl:Mn_0.03_; and comparison tables of related luminescence thermometers (PDF)
