# Supplementary material for: High-Sensitivity and High-Precision Near-Infrared Luminescence Thermometry Using Mn5+-Activated Sr5(VO4)3Cl and Ba5(VO4)3Cl-Based Phosphors
Source: Chem Mater. 2026 Jul 8;38(14):7211–24. doi: 10.1021/acs.chemmater.6c00822 (PMC13420553; doi:10.1021/acs.chemmater.6c00822)
Supplement: Supplementary file 1 [file cm6c00822_si_001.pdf]

High-sensitivity and high-precision near-infrared luminescence thermometry using Mn^{5+}-activated $Sr_5(VO_4)_3Cl$ and $Ba_5(VO_4)_3Cl$-based phosphors

Supplementary Information

Abbi L. Mullins[a], Artemijs Krimovs[a], Zoran Ristić[b], Vesna Djordjević[b], J. A. Gareth Williams[a], Miroslav D. Dramićanin[b] and Ivana Radosavljević Evans[a*]*

[a] *Department of Chemistry, Durham University, Durham, DH1 3LE, UK.*

[b] *Centre of Excellence for Photoconversion, Vinča Institute of Nuclear Sciences National Institute of the Republic of Serbia, University of Belgrade, P.O. Box 522, Belgrade 11001, Serbia.*

**Corresponding author: ivana.radosavljevic@durham.ac.uk*

Table S1: Synthetic conditions for the samples prepared by the solid-state method.

**Chemical formula**	**Mn^{5+} concentration (%)**	**Temperature (°C)(Time (h))**
$Ca_5(VO_4)_3Cl$	0	800(3), 800(3), 800(6), 800(8), 850(12), 850(72)
$Ca_5(V_{0.99}Mn_{0.01}O_4)_3Cl$	1	600(6), 850(12), 850(24), 850(48), 850(36), 850(72), 850(72), 850(72)
$Ca_5(V_{0.97}Mn_{0.03}O_4)_3Cl$	3	
$Ca_5(V_{0.95}Mn_{0.05}O_4)_3Cl$	5	
$Sr_5(VO_4)_3Cl$	0	850(12), 850(12)
$Sr_5(V_{0.99}Mn_{0.01}O_4)_3Cl$	1	850(3), 850(12), 900(6), 850(12)
$Sr_5(V_{0.97}Mn_{0.03}O_4)_3Cl$	3	850(3), 850(12), 850(12)
$Sr_5(V_{0.95}Mn_{0.05}O_4)_3Cl$	5	
$Ba_5(VO_4)_3Cl$	0	850(12), 850(12), 850(12)
$Ba_5(V_{0.99}Mn_{0.01}O_4)_3Cl$	1	800(3), 800(6), 800(24), 850(6), 900(6)
$Ba_5(V_{0.97}Mn_{0.03}O_4)_3Cl$	3	850(3), 850(12), 850(12)
$Ba_5(V_{0.95}Mn_{0.05}O_4)_3Cl$	5	

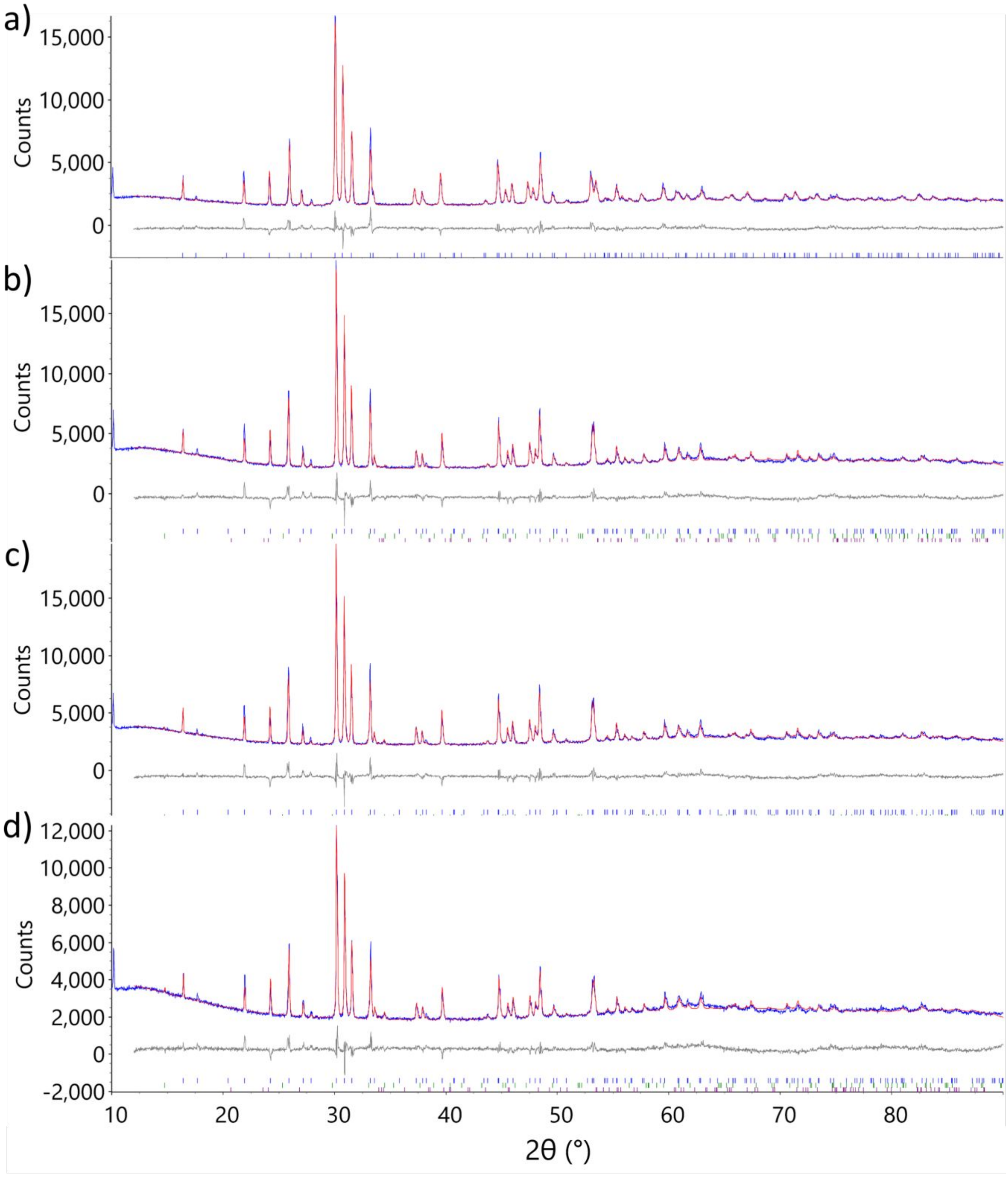

Figure S1: Rietveld fits for materials prepared by the solid-state route: a) $Ca_5(VO_4)_3Cl$, b) $Ca_5(V_{0.99}O_4)_3Cl:Mn_{0.01}$, c) $Ca_5(V_{0.97}O_4)_3Cl:Mn_{0.03}$, d) $Ca_5(V_{0.95}O_4)_3Cl:Mn_{0.05}$.

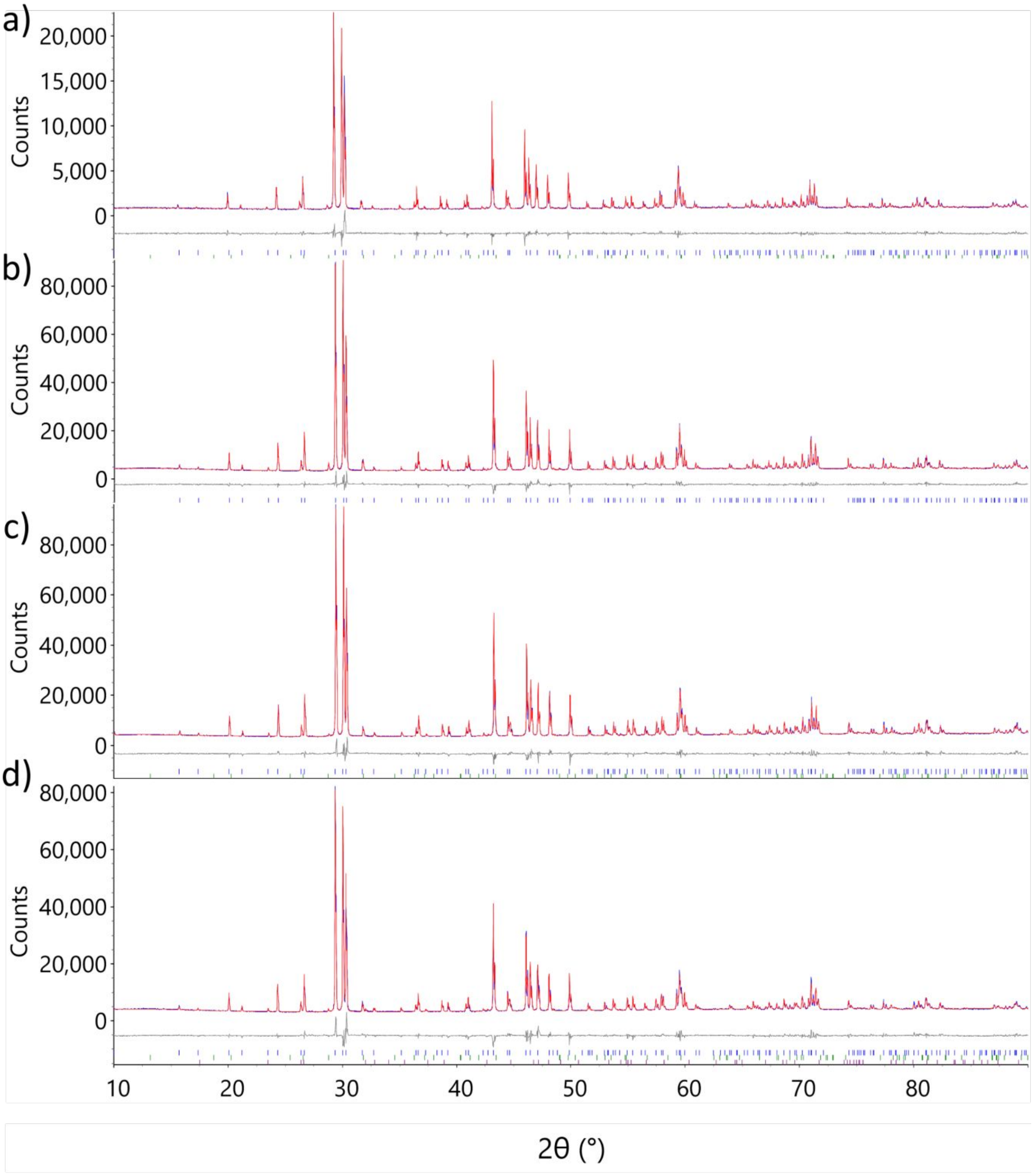

Figure S2: Rietveld fits for materials prepared by the solid-state route: a) $Sr_5(VO_4)_3Cl$, b) $Sr_5(V_{0.99}O_4)_3Cl:Mn_{0.01}$, c) $Sr_5(V_{0.97}O_4)_3Cl:Mn_{0.03}$, d) $Sr_5(V_{0.95}O_4)_3Cl:Mn_{0.05}$.

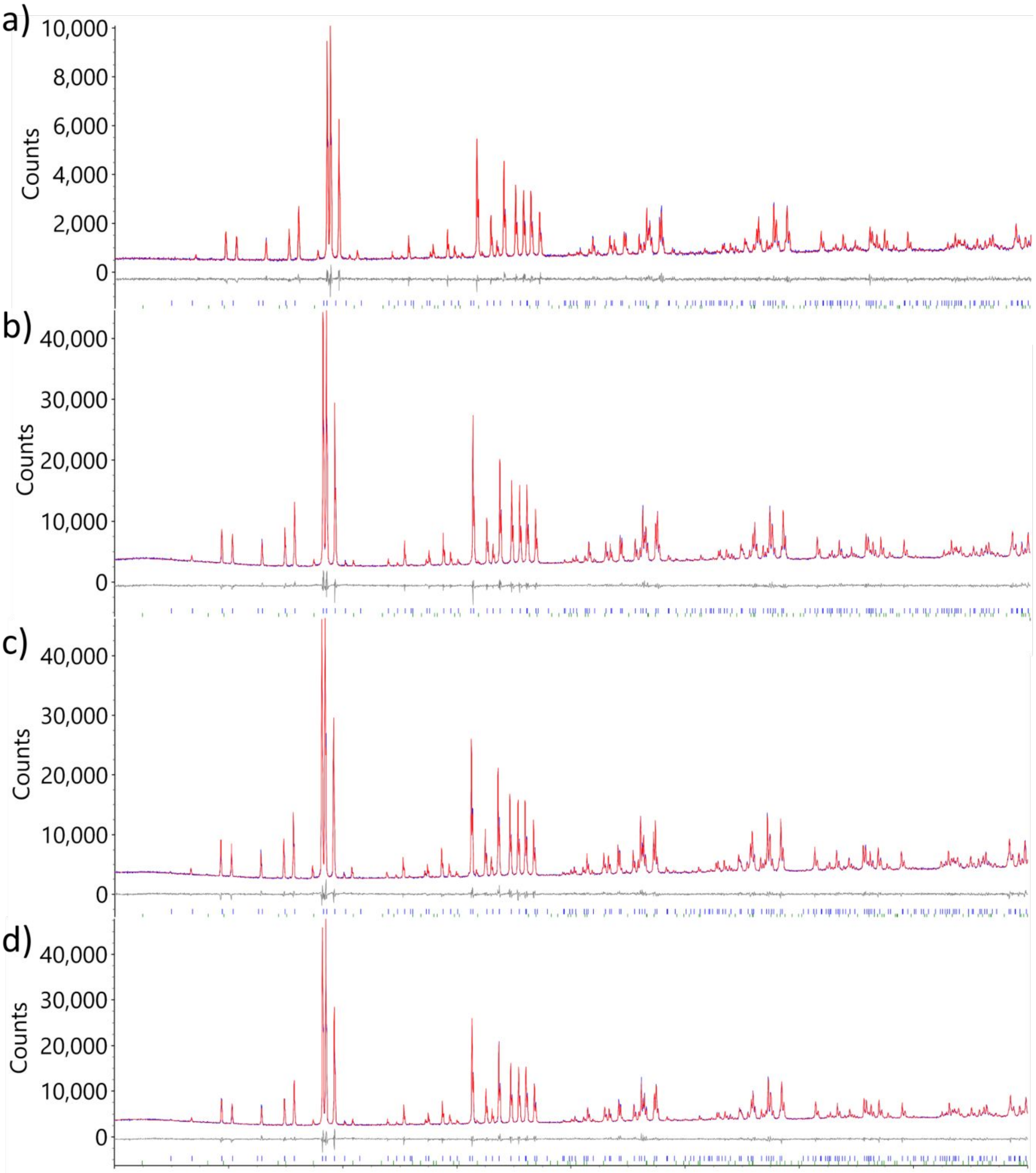

Figure S3: Rietveld fits for materials prepared by the solid-state route: a) $Ba_5(VO_4)_3Cl$, b) $Ba_5(V_{0.99}O_4)_3Cl{:}Mn_{0.01}$, c) $Ba_5(V_{0.97}O_4)_3Cl{:}Mn_{0.03}$, d) $Ba_5(V_{0.95}O_4)_3Cl{:}Mn_{0.05}$.

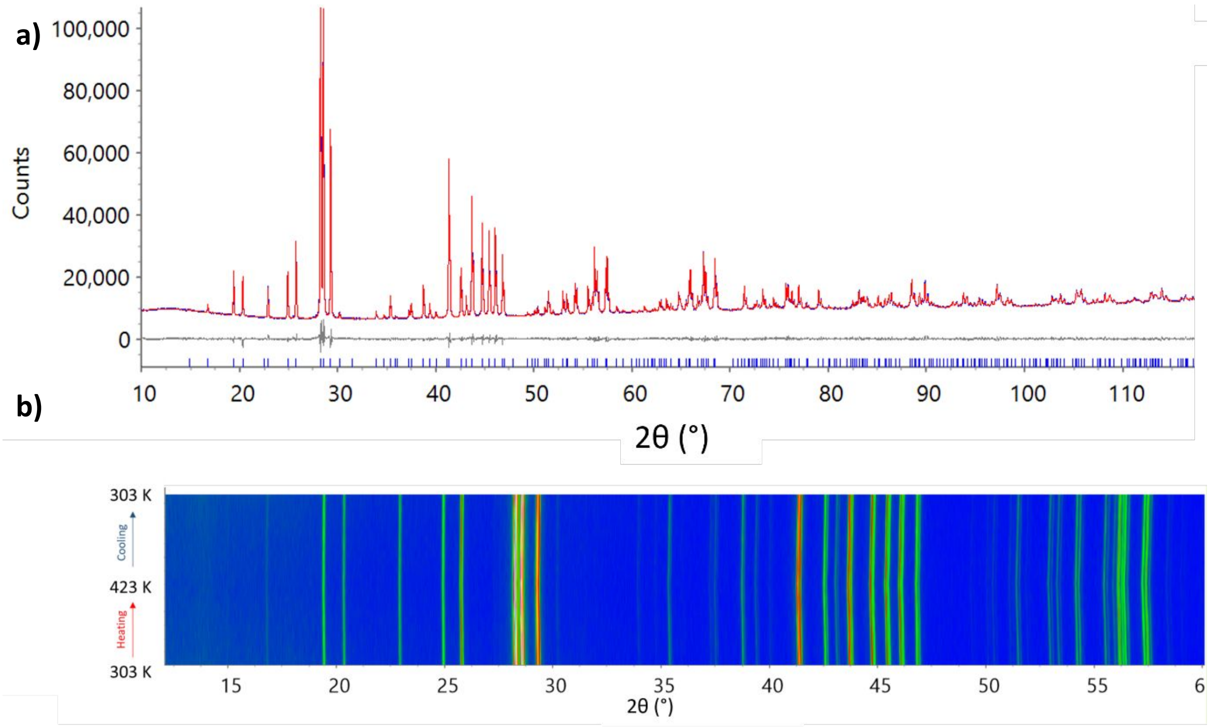

Figure S4a: The final Rietveld fit for $Ba_5(V_{0.97}O_4)_3Cl{:}Mn_{0.03}$ synthesised by the sol-gel route (R_{wp} = 1.99%). The blue curve shows the experimental data, the red curve shows the calculated patterns, and the difference curve is depicted in grey. Tick-marks represent Bragg peak positions, b) Variable temperature PXRD data collected on $Ba_5(V_{0.97}O_4)_3Cl{:}Mn^{5+}_{0.03}$ on heating and cooling.

Table S2: Structural parameters for $Sr_5(V_{0.97}O_4)_3Cl{:}Mn_{0.03}$ and $Ba_5(V_{0.97}O_4)_3Cl{:}Mn_{0.03}$ obtained from Rietveld refinements against PXRD data.

Parameter	$Sr_5(V_{0.97}O_4)_3Cl{:}Mn_{0.03}$	$Ba_5(V_{0.97}O_4)_3Cl{:}Mn_{0.03}$
R_{wp} (%)	3.21	1.99
Unit cell parameter a (Å)	10.20911(9)	10.55442(4)
Unit cell parameter c (Å)	7.30625(7)	7.75600(4)
Unit cell volume V (Å^3)	659.48(1)	748.234(7)
Avg V–O bond length (Å)	1.721(5)	1.717(4)
O–V–O bond angle range (°)	105.4(5) – 113.0(5)	106.4(3) – 113.4(4)
V bond valence sum	5.02(7)	5.05(6)

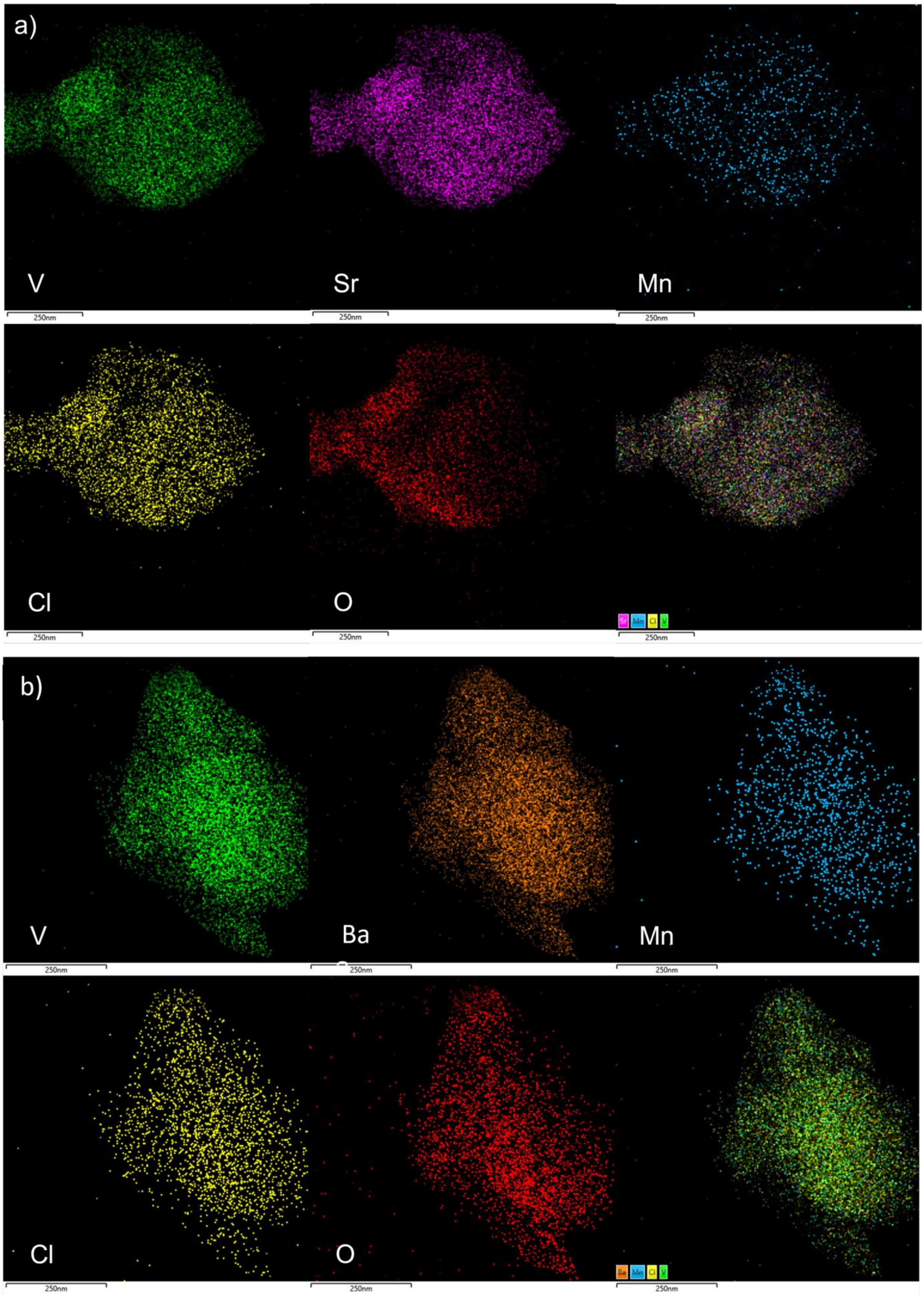

Figure S5: EDX chemical maps of singular grains of a) $Sr_5(V_{0.97}O_4)_3Cl:Mn_{0.03}$, and b) $Ba_5(V_{0.97}O_4)_3Cl:Mn_{0.03}$.

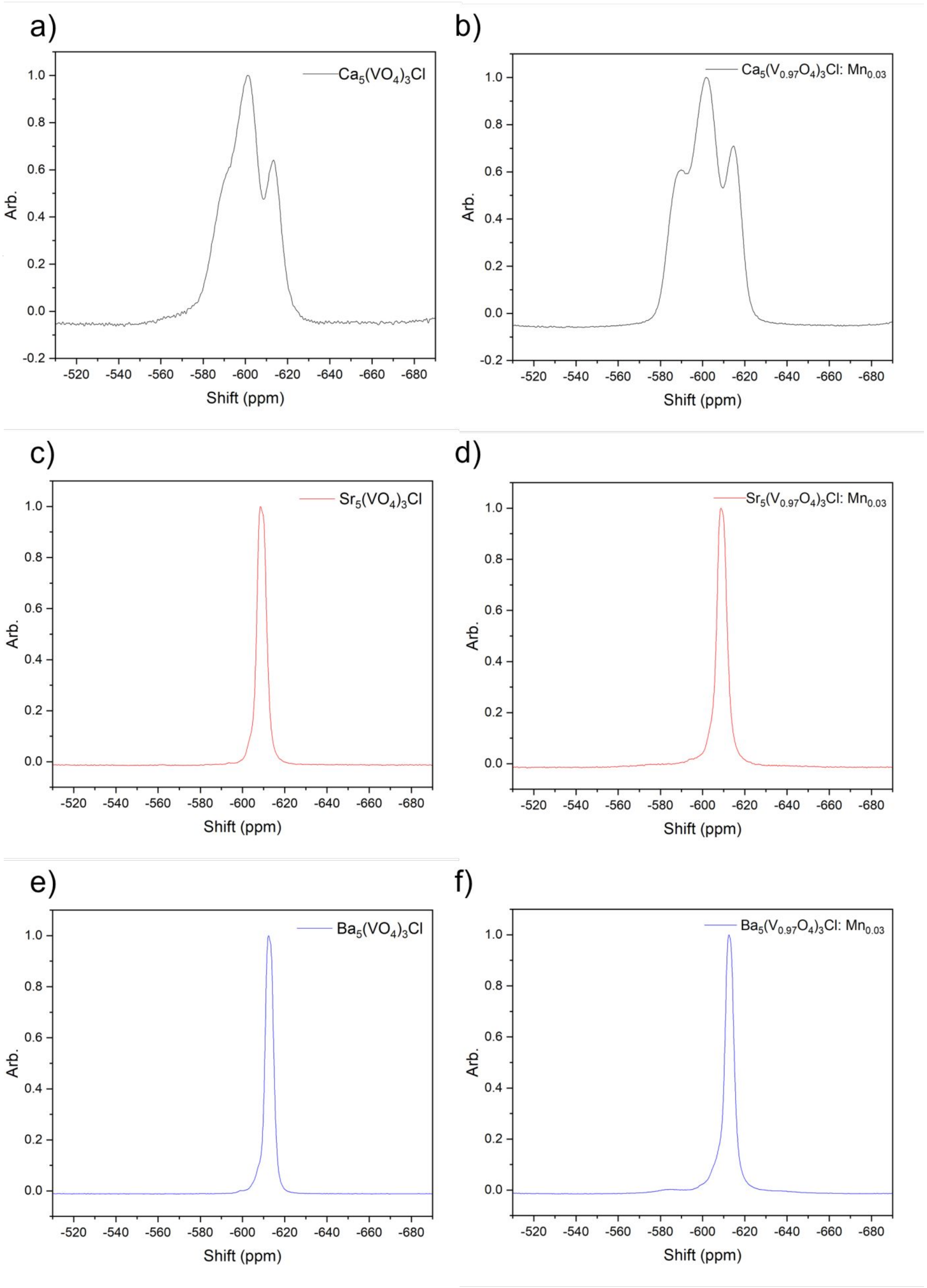

Figure S6: ^{51}V ssNMR spectra acquired for the host materials a) $Ca_5(VO_4)_3Cl$, c) $Sr_5(VO_4)_3Cl$ and e) $Ba_5(VO_4)_3Cl$; and Mn^{5+}-doped materials synthesised by the solid-state route b) $Ca_5(V_{0.97}O_4)_3Cl:Mn_{0.03}$, d) $Sr_5(V_{0.97}O_4)_3Cl:Mn_{0.03}$, and f) $Ba_5(V_{0.97}O_4)_3Cl:Mn_{0.03}$.

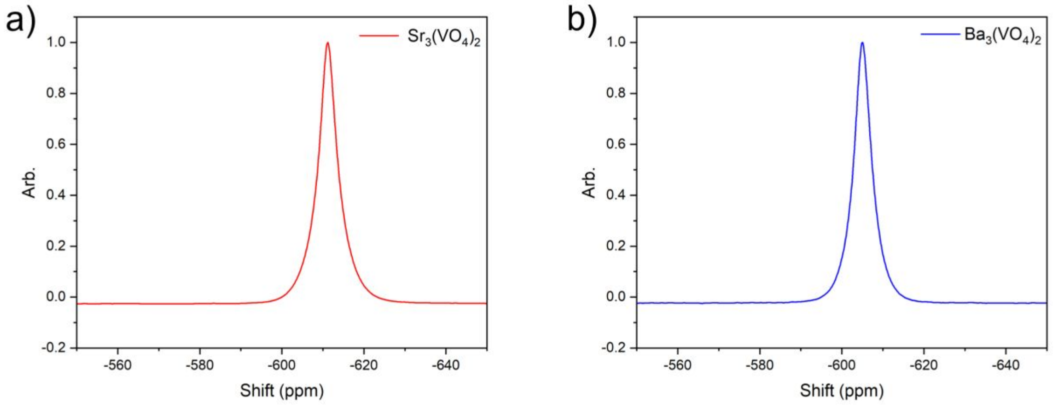

Figure S7: ^{51}V ssNMR of impurities, a) $Sr_3(VO_4)_2$, and b) $Ba_3(VO_4)_2$

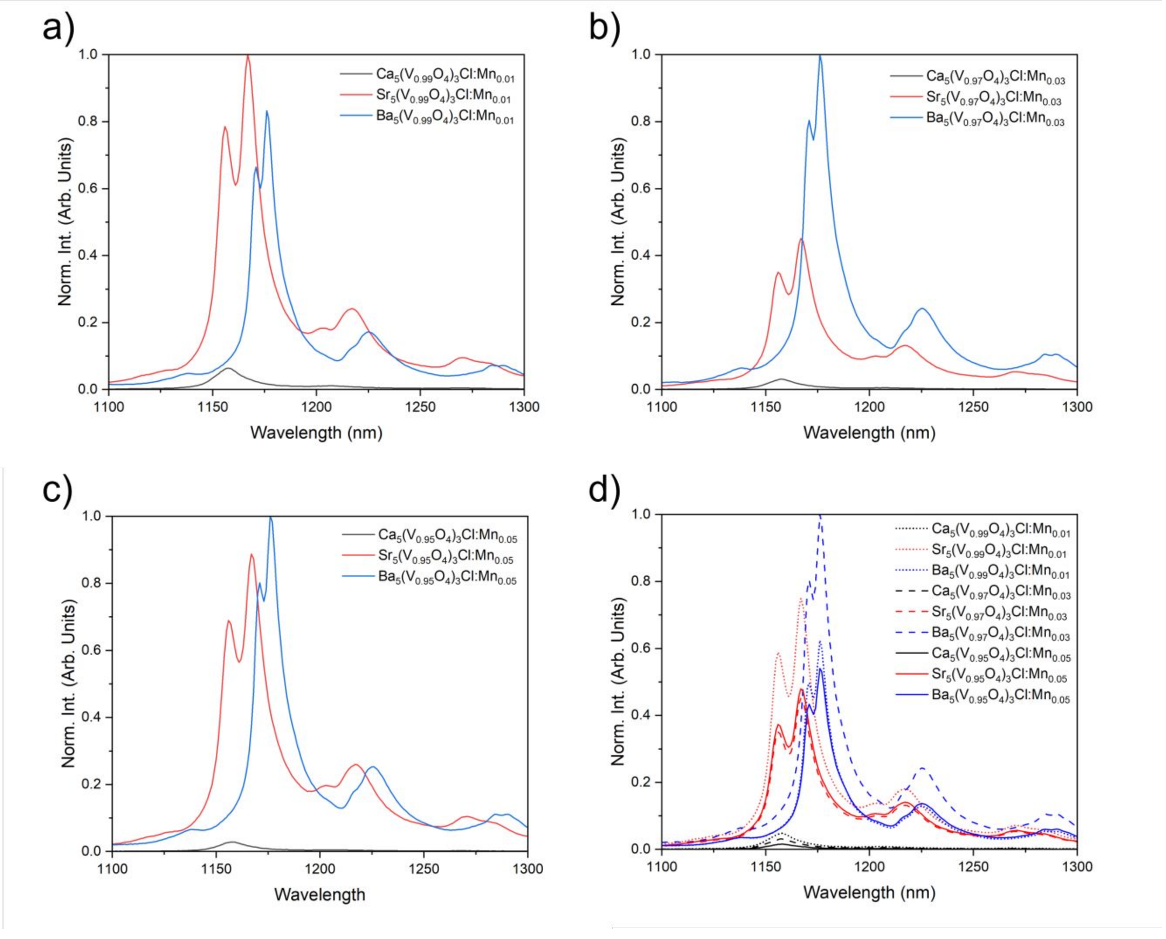

Figure S8: Emission spectra, excited at 700 nm, normalised to highest intensity, of a) $A_5(V_{0.99}O_4)_3Cl:Mn_{0.01}$, b) $A_5(V_{0.97}O_4)_3Cl:Mn_{0.03}$, c) $A_5(V_{0.95}O_4)_3Cl:Mn_{0.05}$, d) all Mn^{5+}-doped samples, where $A^{2+} = Ca^{2+}$, Sr^{2+}, Ba^{2+}.

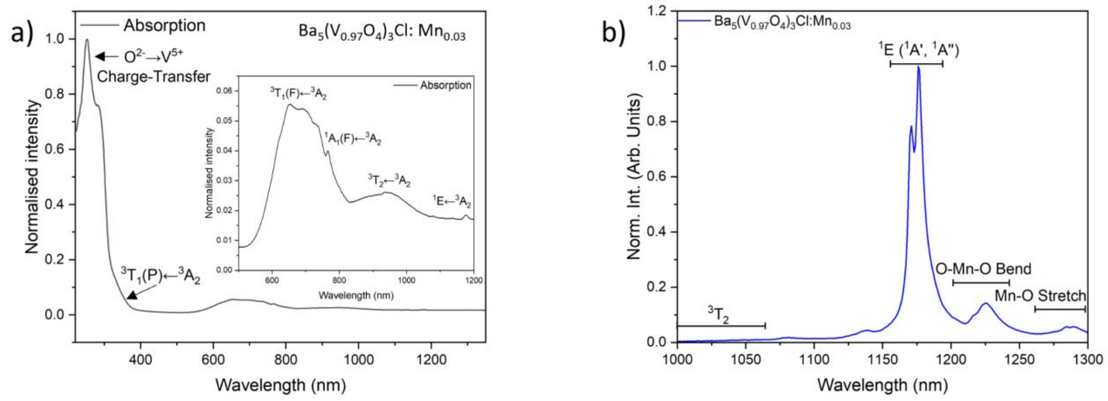

Figure S9: a) Normalised room temperature absorption spectrum (obtained from diffuse reflectance, with inset expansion of the 500-1200 nm region) of $Ba_5(V_{0.97}O_4)_3Cl:Mn_{0.03}$; b) Normalised emission spectrum of $Ba_5(V_{0.97}O_4)_3Cl:Mn_{0.03}$, excited at 700 nm.

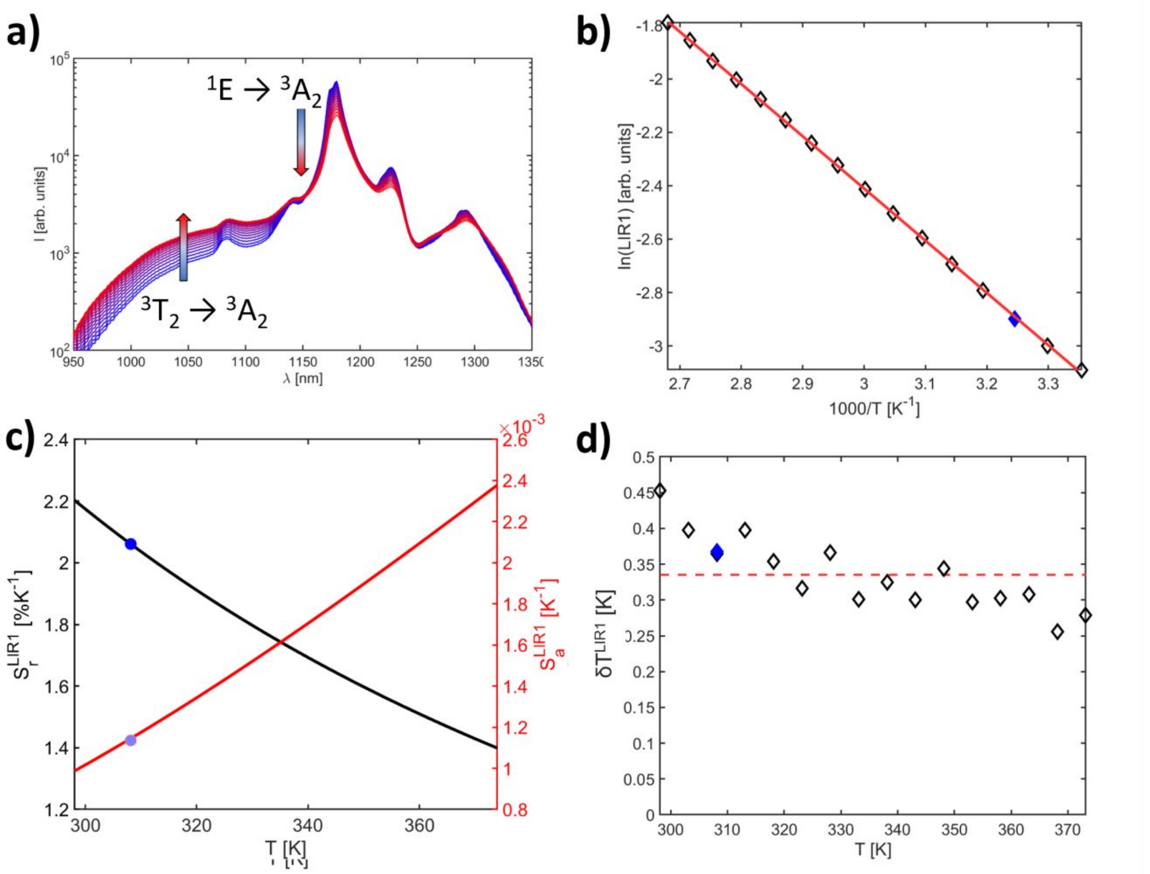

Figure S10: Normalised variable-temperature luminescence data for $Ba_5(V_{0.97}O_4)_3Cl:Mn_{0.03}$: a) Logarithmic-scaled variable-temperature data emission intensity highlighting the changes to intensity of the $^3T_2$ and ^1E bands as temperature increases between 35°C and 100°C; b) The linearised Boltzmann equation, fitting the LIR data (diamond markers – average of 75 scans); c) A plot of the experimentally obtained sensitivities – relative (black, left) and absolute (red, right); d) Temperature resolution of each experimental measurement – the filled blue diamond represents 308 K.

Table S3: Values of the pre-exponential factors and constant obtained by fitting equation (10) to experimental band-shift data for $Sr_5(V_{0.97}O_4)_3Cl:Mn_{0.03}$.

**Fitting Parameters**	**a (K^{-2} cm^{-1})**	**b (K^{-1} cm^{-1})**	**c (cm^{-1})**	**R^2**
**$Sr_5(V_{0.97}O_4)_3Cl$: $Mn_{0.03}$**	3.574 x 10^{-4}	-3.004 x 10^{-1}	8605.62	0.9996

$$E_{MAX}^{Av}(T) = e.T^3 + f.T^2 + g.T + (S1)$$

Table S4: Values of the pre-exponential factors and constant obtained by fitting equation (S1) to experimental band-shift data for $Ba_5(V_{0.97}O_4)_3Cl:Mn_{0.03}$.

**Fitting Parameters**	**e (K^{-3} cm^{-1})**	**f (K^{-2} cm^{-1})**	**g (K^{-1} cm^{-1})**	**h (cm^{-1})**	**R^2**
**$Ba_5(V_{0.97}O_4)_3Cl$: $Mn_{0.03}$**	2.53 x 10^{-6}	1.95 x 10^{-3}	-4.95 x 10^{-1}	8522.80	0.9999

Table S5: Values of the pre-exponential factors and constant obtained by fitting equation (11) to experimental bandwidth data for $Ba_5(V_{0.97}O_4)_3Cl:Mn_{0.03}$.

**Fitting Parameters**	**i (K^{-2} cm^{-1})**	**j (K^{-1} cm^{-1})**	**k (cm^{-1})**	**R^2**
**$Sr_5(V_{0.97}O_4)_3Cl$: $Mn_{0.03}$**	1.996 x 10^{-3}	-8.316 x 10^{-1}	219.34	0.9999
**$Ba_5(V_{0.97}O_4)_3Cl$: $Mn_{0.03}$**	1.70 x 10^{-3}	-7.18 x 10^{-1}	165.43	0.9999

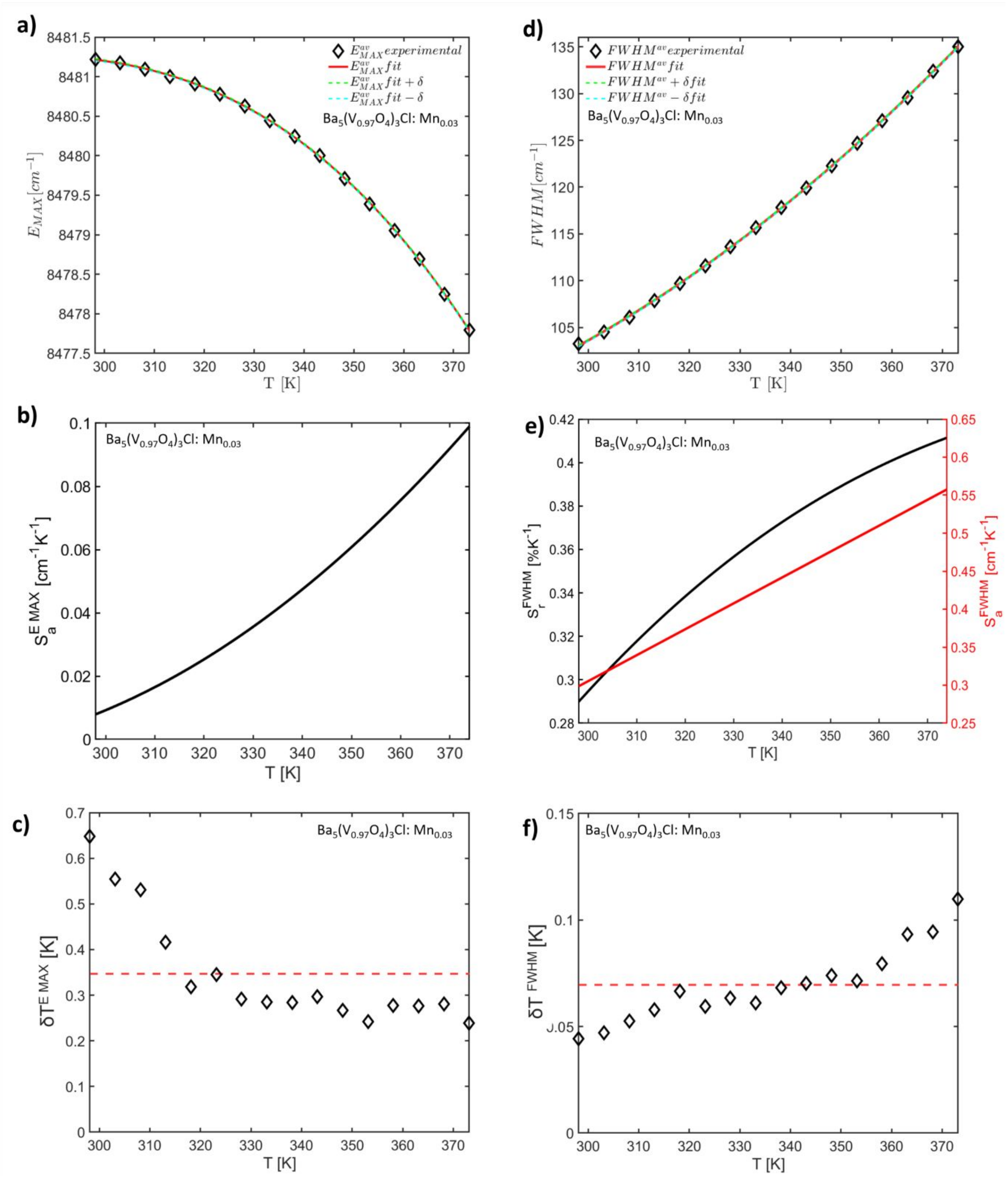

Figure S11: Band-shift thermometry data of samples $Ba_5(V_{0.97}O_4)_3Cl:Mn_{0.03}$. a) Fitted sample peak maxima data as absolute energy (cm^{-1}) with dashed lines showing a 95% confidence interval; b) absolute sensitivity, S_a; c) temperature resolution with average over the temperature range shown by the dashed red line. d) Fitted peak width data as absolute energy (cm^{-1}) with dashed lines showing a 95% confidence interval; e) absolute sensitivity, S_a (red), and relative sensitivity, S_r, (black); f) temperature resolution with average over the temperature range shown by the dashed red line. $Ba_5(V_{0.97}O_4)_3Cl:Mn_{0.03}$, the $^3T_2$ band intensity was integrated between 950-1067 nm and ^1E doublet between 1152-1208 nm.

Table S6: Thermometric figures of merit for materials $Sr_5(V_{0.97}O_4)_3Cl:Mn_{0.03}$ and $Ba_5(V_{0.97}O_4)_3Cl:Mn_{0.03}$

Figure of merit	Read-out method	$Sr_5(V_{0.97}O_4)_3Cl:Mn_{0.03}$	$Ba_5(V_{0.97}O_4)_3Cl:Mn_{0.03}$
**Avg T_{res} (K)**	LIR	0.15	0.34
**Physio T_{res} (K) – 308 K**	LIR	0.16	0.36
**Max S_a - (K^{-1})**	LIR	2.2×10^{-3} (373 K)	2.4×10^{-3} (373 K)
**Physio S_r (K) – 308 K**	LIR	2.23	2.06
**Max S_r (% K^{-1})**	LIR	2.23 (308 K)	2.20 (298 K)
**Avg T_{res} (K)**	Band-shift	0.22	0.35
**Physio T_{res} (K) – 308 K**	Band-shift	0.05	0.52
**Max S_a (cm^{-1} K^{-1})**	Band-shift	0.08 (308 K)	0.10 (373 K)
**Physio S_a (cm^{-1} K^{-1}) – 308 K**	Band-shift	0.08	0.01
**Avg T_{res} (K)**	Bandwidth	0.08	0.07
**Physio T_{res} (K) – 308 K**	Bandwidth	0.04	0.05
**Max S_a (cm^{-1} K^{-1})**	Bandwidth	0.66 (373 K)	0.55 (373 K)
**Max S_r (% K^{-1})**	Bandwidth	0.35 (373 K)	0.42 (373 K)
**Physio S_r (% K^{-1}) – 308 K**	Bandwidth	0.26	0.31
**Physio T_{res} (K) – 308 K**	MLR	0.042	N/A
**Max S_r (% K^{-1})**	MLR	3.14	N/A
**Physio S_r (% K^{-1}) – 308 K**	MLR	3.14	N/A

Table S7: Comparison table of Mn^{5+} luminescence thermometers, their readout methods, and their figures of merit.

Entry	Material	Dopant	Synthesis route (Temp, °C)	Readout	Max S_r (% K^{-1})	Temp. res (K)	Temp (K)	Ref
1	$Sr_5(VO_4)_3Cl$	Mn^{5+}	Sol-gel, 750	LIR	2.23	0.16	308	This work
				Band-shift	S_a = 0.08 cm^{-1} K^{-1}	0.05	308	
				Bandwidth	0.35	0.04	373	
				MLR	3.14	0.042	308	
2	$Ba_5(VO_4)_3Cl$	Mn^{5+}	Sol-gel, 750	LIR	2.20	0.34	298	This work
				Band-shift	S_a = 0.10 cm^{-1} K^{-1}	0.35	373	
				Bandwidth	0.41	0.07	373	
3	$Ca_6BaP_4O_{17}$	Mn^{5+}	Solid-state, 1280	LIR	2.05	0.21	293	[1]
4	$Ca_6BaP_4O_{17}$	Mn^{5+}	Solid-state, 1200	LIR	2.0	0.22	298	[2]
				LIR	0.3	2.82	298	
				Band-shift	S_a = 0.21 cm^{-1} K^{-1}	0.16	298	
5	$Ba_3(PO_4)_2$	Mn^{5+}	Flame aerosol, 800	Single Int.	0.26	<1	283 - 343	[3]
				LIR	0.43	<1	283 - 343	
				Band-shift	S_a =0.022 nm C^{-1}	<1	283 - 343	
6	$Ba_5(PO_4)_3OH$	Mn^{5+}	Flame aerosol, 800	Single Int.	0.81	N/A	283 - 343	[3]
				LIR	0.34	N/A	283 - 343	
				Band-shift	0.51 % C^{-1}	N/A	283 - 343	
7	$Ba_3(VO_4)_2$	Mn^{5+}, Er^{3+}	Pechini, 1000	Lifetime	1.19	0.026	443	[4]
8	$Ba_3(VO_4)_2$	Mn^{5+}, Nd^{3+}	Pechini, 1000	LIR	0.94	N/A	453	[5]
9	$Ba_3(PO_4)_2$	Mn^{5+}	Solid-state, 1200	Lifetime	0.93	N/A	469	[6]
10	$Sr_3(PO_4)_2$	Mn^{5+}	Solid-state, 1200	Lifetime	1.00	N/A	443	[6]
11	$Sr_5(PO_4)_3X$ (X = F, Cl, Br)	Mn^{5+}	Solid-state, 700-1050	LIR_{3T2-1E}	2.91, 1.68, 2.05	N/A	300	[7]
				LIR_{E1-E2}	0.06, 0.13, 0.33	N/A	300	
				Lifetime	0.30, 0.61, 0.61	N/A	300	

Table S8: Comparison table of BW-1-3 luminescence thermometers, their readout methods, and their figures of merit.

Entry	Material	Dopant	Synthesis route (Temp, °C)	Readout	Max Sr (% K^{-1})	Temp. Res (K)	Temp (K)	Ref
**1**	$Sr_5(VO_4)_3Cl$	Mn^{5+}	Sol-gel, 750	LIR	2.23	0.16	308	This Work
				Band-shift	S_a = 0.08 cm^{-1} K^{-1}	0.05	308	
				Bandwidth	0.35	0.04	373	
				MLR	3.14	0.042	308	
**2**	$Ba_5(VO_4)_3Cl$	Mn^{5+}	Sol-gel, 750	LIR	2.20	0.34	298	This Work
				Band-shift	S_a = 0.10 cm^{-1} K^{-1}	0.35	373	
				Bandwidth	0.41	0.07	373	
**3**	YAG	Yb^{3+}, Er^{3+}	Pechini, 1100	LIR	1.0	N/A	163	[8]
				LIR	0.73	N/A	83	
				Band-shift	S_a = 0.09 cm^{-1} K^{-1}	N/A	575	
				Bandwidth	0.40	N/A	249	
				Lifetime	0.73	N/A	154	
**4**	YAG	Yb^{3+}, Er^{3+}	Pechini, 1100	LIR	1.0	N/A	163	[8]
				LIR	0.80	N/A	83	
				Band-shift	S_a = 0.08 cm^{-1} K^{-1}	N/A	575	
				Bandwidth	0.46	N/A	201	
				Lifetime	0.86	N/A	156	
**5**	LaF_3	Nd^{3+}	Co-precipitation, RT	LIR	0.1	2	283 - 343	[9]
				Band-shift	S_a = 0.10 cm^{-1} K^{-1}	2	283 - 343	
**6**	YVO_4	Nd^{3+}	Pechini, 950	LIR	0.54	0.1	303	[10]
				Band-shift	S_a = 0.75 cm^{-1} K^{-1}	17	303	
				Bandwidth	0.14	3	298	
**7**	$CaAl_2O_4$	Co^{3+}, Nd^{3+}	Pechini, 850-1100	LIR	1.43	~0.1	303	[11]
				Lifetime	2.36	N/A	349	
**8**	Ag_2S	N/A	Not provided	Single Int.	5.0	N/A	298	[12]
				LIR	1.4	N/A	298	
				Band-shift	S_a = 1.65 nm C^{-1}	N/A	298	

References:

1. Dramićanin, M. D. *et al.* Mn^{5+}-activated $Ca_6Ba(PO_4)_4O$ near-infrared phosphor and its application in luminescence thermometry. *Light Sci. Appl.* **11**, 279 (2022).
2. Alrebdi, T. A., Alodhayb, A. N., Ristić, Z. & Dramićanin, M. D. Comparison of Performance between Single- and Multiparameter Luminescence Thermometry Methods Based on the Mn^{5+} Near-Infrared Emission. *Sensors* **23**, 3839 (2023).
3. Gschwend, P. M., Niedbalka, D., Gerken, L. R. H., Herrmann, I. K. & Pratsinis, S. E. Simultaneous Nanothermometry and Deep-Tissue Imaging. *Adv. Sci.* **7**, 2000370 (2020).
4. Piotrowski, W. M. *et al.* Mn^{5+} Lifetime-Based Thermal Imaging in the Optical Transparency Windows Through Skin-Mimicking Tissue Phantom. *Adv. Opt. Mater.* **11**, 2202366 (2022).
5. M. Piotrowski, W. *et al.* Critical evaluation of the thermometric performance of ratiometric luminescence thermometers based on $Ba_3(VO_4)_2$:Mn^{5+}, Nd^{3+} for deep-tissue thermal imaging. *J. Mater. Chem. C* **11**, 6713–6723 (2023).
6. Ristić, Z. *et al.* Near-Infrared Luminescent Lifetime-Based Thermometry with Mn^{5+}-Activated $Sr_3(PO_4)_2$ and $Ba_3(PO_4)_2$ Phosphors. *ACS Appl. Electron. Mater.* **4**, 1057–1062 (2022).
7. Back, M. *et al.* Anion Effect on the Thermometric Properties of Near-Infrared Emitting Mn5+-Activated Sr5(PO4)3X (X = F, Cl, Br) Apatite Phosphors. *Adv. Opt. Mater.* **14**, e03109 (2026).
8. Periša, J. *et al.* All near-infrared multiparametric luminescence thermometry using Er^{3+}, Yb^{3+}-doped YAG nanoparticles. *RSC Adv.* **11**, 15933–15942 (2021).
9. Rocha, U. *et al.* Subtissue Thermal Sensing Based on Neodymium-Doped LaF_3 Nanoparticles. *ACS Nano* **7**, 1188–1199 (2013).
10. Kolesnikov, I. E. *et al.* YVO_4:Nd^{3+} nanophosphors as NIR-to-NIR thermal sensors in wide temperature range. *Sci. Rep.* **7**, 18002 (2017).
11. Kniec, K., Kochanowska, A., Li, L., Suta, M. & Marciniak, L. A ratiometric and lifetime-based luminescent thermometer exploiting the Co^{3+} luminescence in $CaAl_2O_4$:Co^{3+} and $CaAl_2O_4$:Co^{3+},Nd^{3+}. *J. Mater. Chem. C* **10**, 9278–9286 (2022).

12. Shen, Y. *et al.* Ag_2S Nanoheaters with Multiparameter Sensing for Reliable Thermal Feedback during In Vivo Tumor Therapy. *Adv. Funct. Mater.* **30**, 2002730 (2020).
